# Initial Red Cell Infusion Rate and Escalation of Respiratory Support in Critically Ill Adults: A Target Trial Emulation

**DOI:** 10.3390/jcm15208147

**Published:** 2026-10-20

**Authors:** Zhenkun Zhao, Xiaoye Chen, Zhongjing Pan, Ao Sha, Yadong Zhou, Xiaoli Chen, Tao Cheng, Lu Gan

**Affiliations:** 1 Department of Emergency Medicine, West China Hospital, Sichuan University, Chengdu 610041, China; zhaozhenkun@stu.scu.edu.cn (Z.Z.);; 2 Department of General Surgery, West China Tianfu Hospital, Sichuan University, Chengdu 610041, China; 3 West China School of Nursing, West China Hospital, Sichuan University, Chengdu 610041, China

**Keywords:** red blood cell transfusion, infusion rate, critical care, respiratory support, target trial emulation

## Abstract

**Background/Objectives:** Current transfusion guidelines address when red cells should be given, not how fast. We estimated the association between the initial infusion rate of a first red cell transfusion and escalation of respiratory support in critically ill adults. **Methods:** We emulated a target trial in MIMIC-IV v3.1. Of 2790 eligible adults, 1787 received a slower (50–175 mL/h) and 1003 a faster (>175–350 mL/h) initial rate; the primary outcome was escalation to advanced respiratory support or death within 12 h. Overlap weighting estimated marginal risk differences in the overlap population, with 2000 bootstrap resamples refitting the propensity-score model. Sensitivity and continuous dose–response analyses were pre-specified. **Results:** The primary outcome was classifiable in 2671 patients, with 143 events. Weighted 12 h risks were 4.95% and 6.60%: risk difference 1.65 percentage points (95% CI −0.41 to 3.74), risk ratio 1.33 (0.93 to 1.92). Invasive mechanical ventilation or death, a nested key secondary outcome unadjusted for multiplicity, gave 2.55 percentage points (0.71 to 4.59); 28-day mortality gave −1.45 (−4.10 to 1.30). In the pre-specified dose–response analysis, standardised 12 h risk was lowest near 140 mL/h (4.27%) and rose to 8.83% (6.56 to 11.28) at 350 mL/h. **Conclusions:** The primary analysis did not distinguish the two initial-rate strategies, although the interval remained compatible with a clinically important increase in risk. The secondary and dose–response findings are hypothesis-generating and do not support changing current infusion-rate practice. These estimates support the design and sample-size planning of a prospective trial assigning infusion rate.

## 1. Introduction

Red blood cell (RBC) transfusion is a common supportive therapy in critical care. In an international prospective cohort of 3643 adults in 233 intensive care units (ICUs), 25% received at least one RBC transfusion, and both the proportion transfused and the haemoglobin (Hb) level preceding transfusion varied markedly across centres [1]. Over the past two decades, randomised trials have focused largely on when RBCs should be given, and the guidelines derived from them recommend restrictive Hb-based transfusion thresholds for most haemodynamically stable hospitalised patients [2,3,4]. Once the decision to transfuse has been taken, however, there is little outcome-based evidence to guide how fast an RBC unit should be administered.

RBC infusion rate is a modifiable component of transfusion practice with plausible physiological consequences [5,6]. In routine care, the initial rate is selected and subsequently adjusted by the bedside clinical team [7], yet outcome-based evidence to guide rate selection remains limited [8,9]. Transfusion-associated circulatory overload (TACO) is among the most frequent serious adverse transfusion reactions and an important cause of transfusion-related morbidity and mortality, and infusion rate has been identified as one of its risk factors [5,8,9,10]. Current guidance also specifies that RBC transfusion should be completed within four hours of removal from temperature-controlled storage, a limit derived from bacterial proliferation and product quality rather than from evidence on patient outcomes [7,11]. Experimental work further supports a haemodynamic effect of rate itself: in anaemic rats with acute myocardial infarction, faster RBC transfusion produced a greater rise in left ventricular end-diastolic pressure [6]. Together, these findings give the question biological and clinical plausibility, but the available evidence derives largely from observational studies of TACO and from animal experiments, and does not establish whether the initial RBC infusion rate is associated with subsequent escalation of respiratory support.

Infusion rate may be individualised to illness severity, haemodynamic status, and perceived volume tolerance and adjusted as clinical conditions evolve, complicating randomised comparisons in critically ill patients [5,7,10]. Target trial emulation specifies the treatment strategies, eligibility criteria, time zero, follow-up, and outcomes of the trial that would answer a clinical question, then maps these elements onto observational data [12,13,14,15]. It offers a structured framework when a randomised trial is impractical and helps avoid design bias arising when eligibility, treatment assignment, and the start of follow-up are not aligned; the approach has been applied to comparable questions in intensive care [16,17,18]. We therefore emulated a target trial using MIMIC-IV to compare a slower (50–175 mL/h) with a faster (>175–350 mL/h) initial infusion-rate strategy for the first RBC transfusion in the ICU, with respect to escalation of advanced respiratory support or death within 12 h.

## 2. Materials and Methods

### 2.1. Study Design and Reporting

We emulated a target trial comparing two initial infusion-rate strategies for red cell transfusion in intensive care. Protocol, code, run log, and results are registered at https://osf.io/cmeys/ (accessed on 18 August 2026) (DOI 10.17605/OSF.IO/CMEYS). Reporting follows the TARGET statement [19]; item-by-item correspondence is given in the TARGET checklist submitted with this manuscript, and the target trial protocol is Table 1.

### 2.2. Data Source and Participants

We used MIMIC-IV v3.1, a de-identified critical care database covering 2008–2022 from a single US academic medical centre, under a credentialed data use agreement; collection of the source data was reviewed by the institutional review board at Beth Israel Deaconess Medical Center, which granted a waiver of informed consent [20,21].

Adults were eligible at the first recorded RBC transfusion episode during the ICU stay in which they first received RBCs, provided that the initial infusion rate was ≥50 mL/h and that time zero occurred at least 4 h after ICU admission. Episodes were excluded if (1) any of six pre-specified, high-specificity indicators of active bleeding or a bleeding-prone clinical context were identified using the specified ascertainment windows; (2) invasive mechanical ventilation was already being delivered at baseline, precluding further escalation on the respiratory support scale; or (3) the baseline respiratory support level could not be reliably determined.

The antecedent clinical-record indicators used specified look-back windows; trauma was identified from hospitalisation-coded diagnoses whose presence and availability at time zero could not be verified. The concurrent-transfusion indicator used a co-initiation window from the episode start to less than 15 min afterwards, flagging more than one RBC record or the start of another blood component. This was an operational proxy for concurrent transfusion rather than a verified count of simultaneously transfused units; records within a single episode can also represent segmentation of the infusion.

### 2.3. Treatment Strategies and Time Zero

The target trial and its emulation are set out in Table 1. Strategies were defined by the rate on the first line of the first episode: slower, 50–175 mL/h, and faster, >175–350 mL/h. The cut-points correspond to a 350 mL unit given over two hours and over one hour rather than to distributional quantiles. Episodes above 350 mL/h were excluded from the binary-strategy comparison but retained in the separately constructed cohort for the pre-specified dose–response analysis. The comparison assumes that this first recorded rate reflects the strategy implemented at time zero; later rate changes did not reclassify patients, and the rate achieved over the whole infusion was not used as an exposure because it is knowable only after follow-up has begun (Supplementary Methods S1). Accordingly, this comparison concerns the initial recorded strategy with subsequent clinically indicated adjustments permitted, rather than maintenance of a specified rate throughout transfusion. The database rate was not independently validated against pump telemetry.

**Figure 1:**
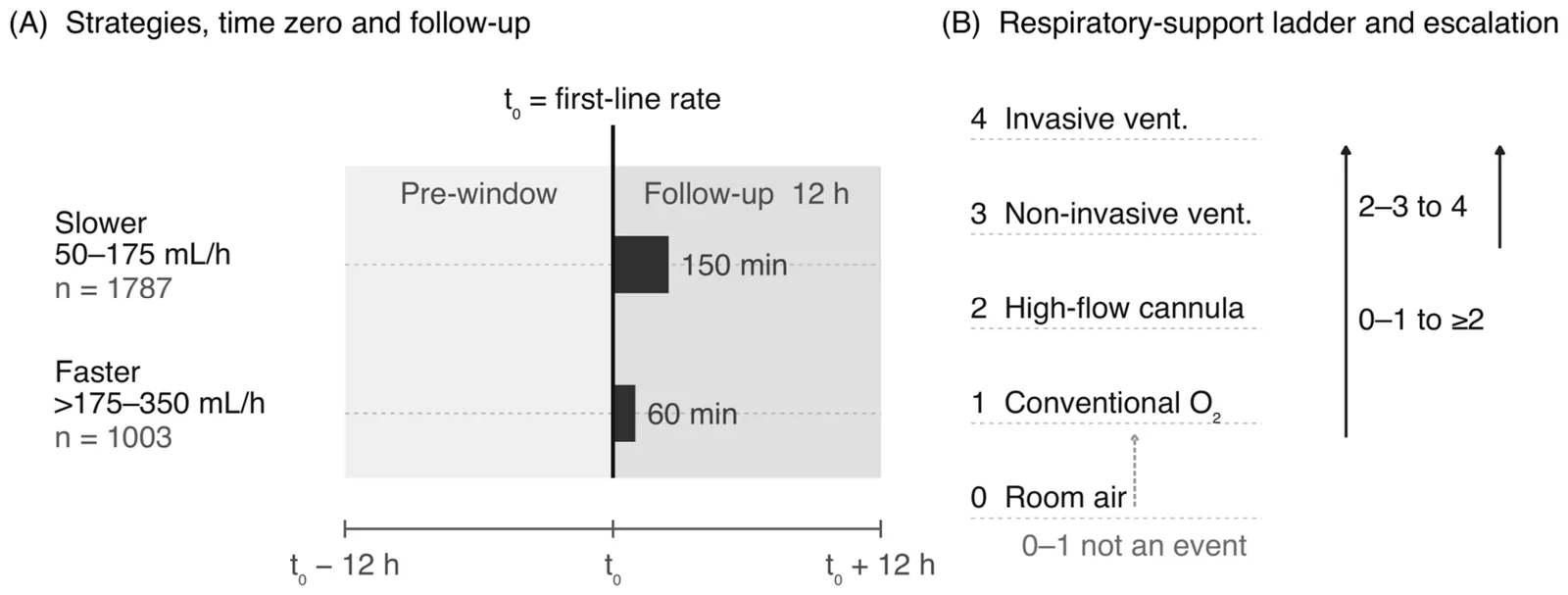
Study design, initial-rate strategies and respiratory support outcome. (**A**) Time zero was the start of the first RBC transfusion episode in intensive care. (**B**) Respiratory support was graded from room air/no supplemental oxygen (level 0) to invasive mechanical ventilation (level 4). In panel B, solid arrows indicate qualifying escalation to advanced respiratory support: from baseline levels 0–1 to level ≥2, or from baseline levels 2–3 to level 4. The grey dashed arrow indicates a transition from level 0 to level 1, which does not meet the primary respiratory-escalation criterion.

Time zero was the start of that episode, and the initial recorded rate defined strategy classification at the start of follow-up, with no treatment-assignment grace period (Figure 1A). Time-stamped clinical covariates were anchored at or before time zero. The Charlson index, heart failure and chronic kidney disease were hospitalisation-code-derived proxies whose presence and availability at time zero could not be verified.

### 2.4. Outcomes and Ascertainment

Respiratory support was represented on a five-level scale (Figure 1B): room air/no supplemental oxygen (0), conventional oxygen (1), high-flow nasal cannula (2), non-invasive ventilation (3), and invasive mechanical ventilation (4). The primary outcome was escalation of advanced respiratory support or death within 12 h. For patients at baseline levels 0–1, escalation was defined as new high-flow nasal cannula, non-invasive ventilation, or invasive mechanical ventilation [22]; for those at baseline levels 2–3, escalation was defined as new invasive mechanical ventilation. The outcome was analysed as a binary 12 h endpoint rather than as a time-to-event outcome.

Two key secondary outcomes were pre-specified as supporting analyses without adjustment for multiplicity: (1) an increase of at least one respiratory support level or death and (2) invasive mechanical ventilation or death. Other pre-specified secondary outcomes, including 28-day mortality, are reported in Table S8.

Respiratory support was ascertained from four electronic record sources using a single pre-specified algorithm. Three sources directly contributed to respiratory-level assignment, whereas airway-procedure records contributed to ascertainment without independently assigning a level. Confirmed absence of supplemental oxygen was classified as non-escalation rather than as missing information. Death was ascertained hospital-wide, whereas respiratory support was observable only during ICU care. Detailed level-assignment rules, handling of unclassifiable outcomes and ward transfers, and corresponding sensitivity analyses are provided in Supplementary Methods S2 and Table S1. For patients transferred to a general ward before 12 h without a previously observed event, the primary rule assumed no subsequent respiratory escalation; this was an assumption rather than direct post-transfer observation. The pre-specified S24 analysis excluded these patients, while retaining the original propensity-score model and weights.

The primary endpoint was pre-specified as a pragmatic measure of early respiratory deterioration that could be reproducibly ascertained from the available electronic health record data. Adjudicated transfusion-associated circulatory overload could not be reliably reconstructed from structured MIMIC-IV data and was therefore not used as an outcome [23,24,25]. Respiratory support escalation should consequently be interpreted as a clinical support outcome. The endpoint classified each patient as having or not having an event; the support ladder defined the event rather than serving as an ordinal outcome. The baseline-dependent thresholds sought to distinguish escalation to advanced support from initiation of conventional oxygen alone. Death was included to avoid classifying a patient who died before escalation as having a favourable respiratory outcome. Nevertheless, the components differ in severity, support escalation reflects clinical decisions, and death is not specific to respiratory deterioration.

### 2.5. Statistical Analysis

The primary estimand was the marginal 12 h risk difference, faster minus slower, in the population targeted by overlap weighting, which gives greatest weight to patients with empirical equipoise between the two initial-rate strategies. Risk ratios were reported as complementary relative effect measures. Propensity scores for receipt of the faster strategy were estimated using logistic regression, and overlap weights were applied [26]. The pre-specified adjustment set included demographic characteristics, admission context, illness severity, comorbidities, renal and haemodynamic status, antecedent fluid exposure, baseline respiratory support, transfusion-unit characteristics, workflow variables, and baseline haemoglobin. Continuous covariates were modelled using restricted cubic splines with knots at the 5th, 35th, 65th, and 95th percentiles. Covariate definitions and model specifications are provided in Supplementary Methods S3 and S4.

RBC storage duration was not included because collection and expiry dates or a storage-age field were not available in the structured transfusion records used for this analysis. The “inputevents storetime” field represents documentation or validation time, not the storage age of the blood component.

A three-tier missing-data rule was specified before missingness was quantified. Lactate, although pre-specified as a candidate covariate, was excluded from the primary propensity-score model under this rule because 67.7% of values were missing; no patient was excluded because of missing baseline covariates. The propensity-score model was fitted in the full cohort of 2790 patients, and the resulting weights were then applied to the relevant outcome-analysis population rather than refitting the model after restriction by outcome ascertainability. Covariate balance was assessed using standardised mean differences and propensity-score overlap, with additional distributional diagnostics reported in Supplementary Methods S4 and Figures S1, S2 and S4. Overlap weights were not truncated because they are bounded by construction.

Causal interpretation of the weighted contrasts requires consistency, conditional exchangeability given the measured baseline covariates, and positivity within the overlap population. These assumptions are not guaranteed by weighting; overlap weighting was used to adjust for measured baseline confounding and to reduce reliance on observations with limited empirical treatment overlap. Assumptions concerning outcome ascertainment and classification are described in Supplementary Methods S2.

Marginal risks under each strategy were estimated using the overlap-weighted sample, from which risk differences and risk ratios were derived. Uncertainty was quantified using 95% percentile bootstrap confidence intervals from 2000 non-parametric resamples of ICU stays. In each resample, the propensity-score model was refitted, overlap weights were recomputed, the relevant analysis population was applied, and the effect measures were re-estimated.

Twenty-six sensitivity analyses were pre-specified in the locked protocol; 25 were conducted because the pre-specified trigger for multiple imputation was not met (Supplementary Methods S5). These analyses examined alternative exposure and outcome definitions, weighting specifications, eligibility restrictions, covariate definitions, outcome ascertainment, and other design and analytic choices. Pre-specified falsification analyses applied the respiratory-outcome algorithm to the 12 and 6 h periods before time zero using corresponding pseudo-baselines; because pseudo-baseline ascertainment and look-back completeness were not available for all episodes, these analyses were restricted to eligible ascertainable populations as specified in Supplementary Methods S2 and Table S5. A further pre-specified negative-control outcome was a new positive culture occurring 72 h to 7 days after time zero [27,28]. E-values were calculated for the risk ratio point estimate and the confidence limit closest to the null [29].

Five subgroups were pre-specified as exploratory, without adjustment for multiplicity. Before examination of subgroup effect estimates, quantitative reporting was suppressed when either strategy represented <5% of a stratum or either strategy group contained fewer than 25 outcome events. Analyses not specified in the locked protocol are explicitly labelled as post hoc and interpreted descriptively.

A post hoc descriptive comparison contrasted all included patients with the other ICU RBC recipients in the source population (Table S10). To provide a common reference point, age and admission characteristics were described at admission to the first ICU stay in which RBCs were recorded. This comparison used no hypothesis testing, outcome comparisons or reweighting and was separate from the pre-specified outcome analyses.

### 2.6. Declaration of AI Use

During the preparation of this work, the authors used Claude Opus 5.0 for the purposes of language editing and debugging the analysis code. The authors have reviewed and edited the output and take full responsibility for the content of this publication.

## 3. Results

### 3.1. Cohort and Transfusion Characteristics

**Figure 2:**
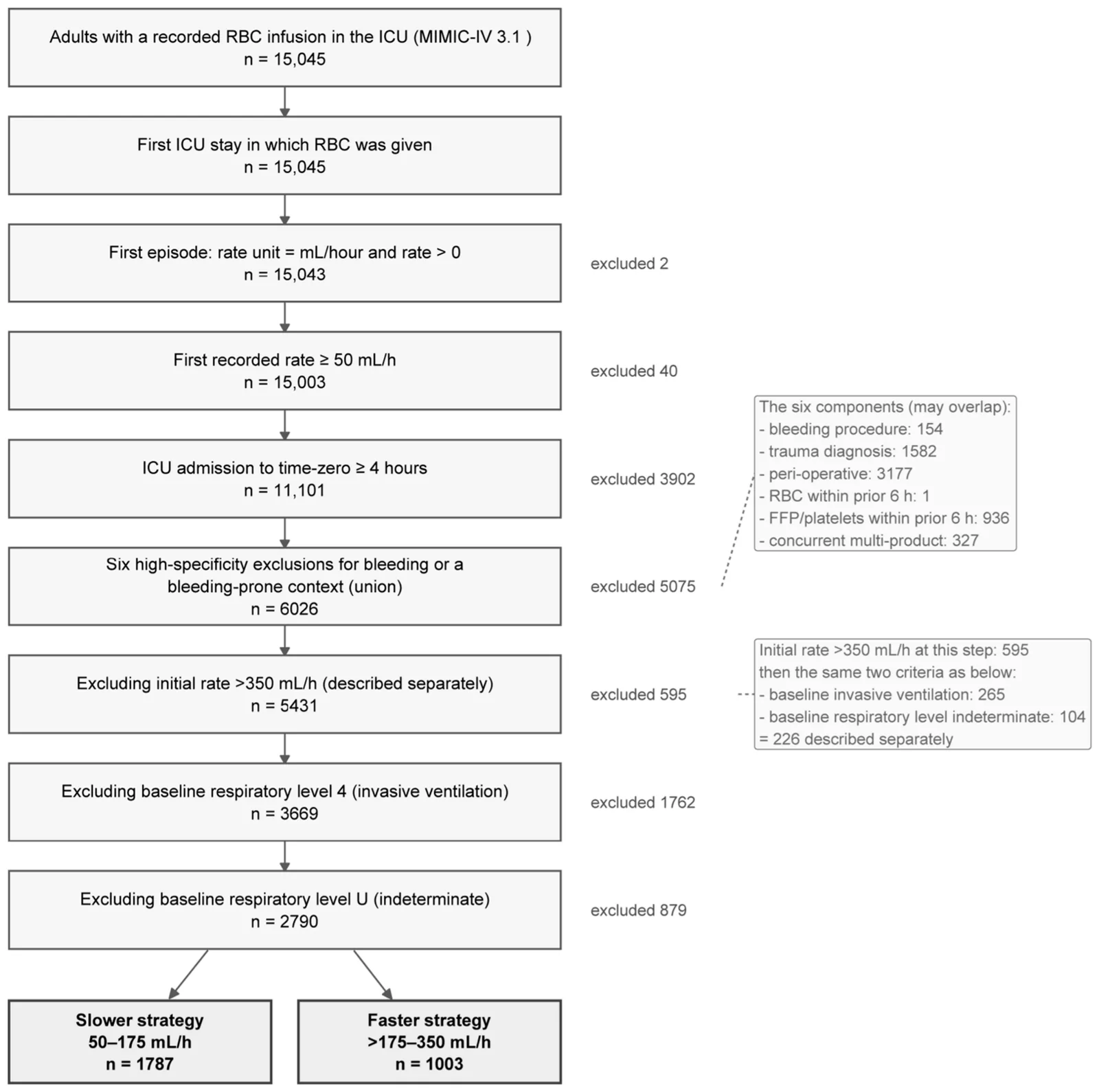
Cohort assembly. Stepwise derivation of the analytic cohort from all adults with a recorded red blood cell transfusion in the intensive care unit. Numbers shown to the right of each box indicate the number of patients excluded at that step.

Of 15,045 adults with a recorded RBC infusion in intensive care, 2790 entered the target-strategy comparison: 1787 in the slower and 1003 in the faster strategy. An additional 226 episodes with initial rates above 350 mL/h met the remaining respiratory eligibility criteria but fell outside the strategy comparison (Figure 2). They are described in Table S6 and contribute only to the pre-specified dose–response analysis. The recorded starting unit volume differed little between strategies (338.0 vs. 339.5 mL; Table 2, with the complete variable list in Table S6).

In the post hoc descriptive comparison, included patients had a median ICU-admission age of 69 years (IQR 58–78), compared with 68 (56–78) among non-included recipients; 47.3% and 44.2% were female. The first recorded RBC infusion occurred a median of 28.98 h (11.22–50.24) versus 9.75 (2.72–30.56) after ICU admission. Care unit composition also differed: coronary care accounted for 13.9% versus 5.9%, and trauma intensive care for 5.7% versus 14.7% (Table S10).

Whole-episode hang time was a median of 150 min (IQR 120–180) under the slower and 60 (60–90) under the faster strategy; multiple infusion-record segments occurred in 200/1787 slower-strategy episodes (11.2%) and 6/1003 faster-strategy episodes (0.6%). Segmentation may reflect a changed recorded rate or interruption, but does not by itself establish a change in delivered flow or a switch between the two strategy ranges. Fifty-two slower-strategy episodes (2.91%) exceeded four hours, compared with none in the faster strategy. In a post hoc descriptive process audit, 123 slower-strategy episodes (6.9%) began below the minimum rate required to deliver their recorded starting unit volume within four hours; no faster-strategy episode did so. Of those 123, 48 exceeded four hours, 59 were documented as completed within four hours, all 59 after an upward rate adjustment, and 16 ended within four hours charted as stopped or paused (Figure S5; Supplementary Methods S6).

### 3.2. Weighting and Outcome Classification

Propensity-score distributions showed moderate empirical overlap (overlap coefficient 0.55), with 0.90% of patients having scores below 0.05 or above 0.95. As expected with overlap weighting, model terms were balanced to within 0.001 on the scale entered in the propensity-score model. The largest residual standardised mean difference on the displayed raw scale was 0.042, for crystalloid volume, which enters the model log-transformed. Distributional diagnostics nevertheless identified residual imbalance in the variance of crystalloid volume per kilogram (Figures S1, S2 and S4).

The pre-specified classification rule included 2671 patients (95.73%) in the primary analysis, with similar classification proportions in the slower and faster strategies (95.75% and 95.71%). The classification was based on directly observed information in 2301 patients (82.47% of all 2790 included patients); the remaining 370 (13.26% of all included patients, or 13.85% of the 2671 patients with classified outcomes) were classified under the ward-transfer assumption rather than by observed post-transfer respiratory support (Table S3). Strategy distribution was similar between patients with classified and unclassified primary outcomes (35.9% vs. 36.1% faster; Table S2). None of the patients transferred to a general ward returned to intensive care within the 12 h window.

In a post hoc check of the outcome, made without reference to the infusion-rate strategy, 28-day in-hospital mortality was 35.7% among the 143 patients with the primary outcome and 9.8% among the 2528 without it. For the escalation-only definition, 28-day in-hospital mortality was 31.3% versus 10.1%; patients who died within 12 h were retained in this comparison. In a separate analysis excluding patients who died within 12 h, mortality rose with the level of support reached: 34.3% in the 99 patients who reached invasive ventilation, 15.6% in the 32 whose escalation stopped at high-flow or non-invasive support, and 9.9% in the 2375 with no escalation (Table S9).

### 3.3. Primary and Key Secondary Outcomes

**Figure 3:**
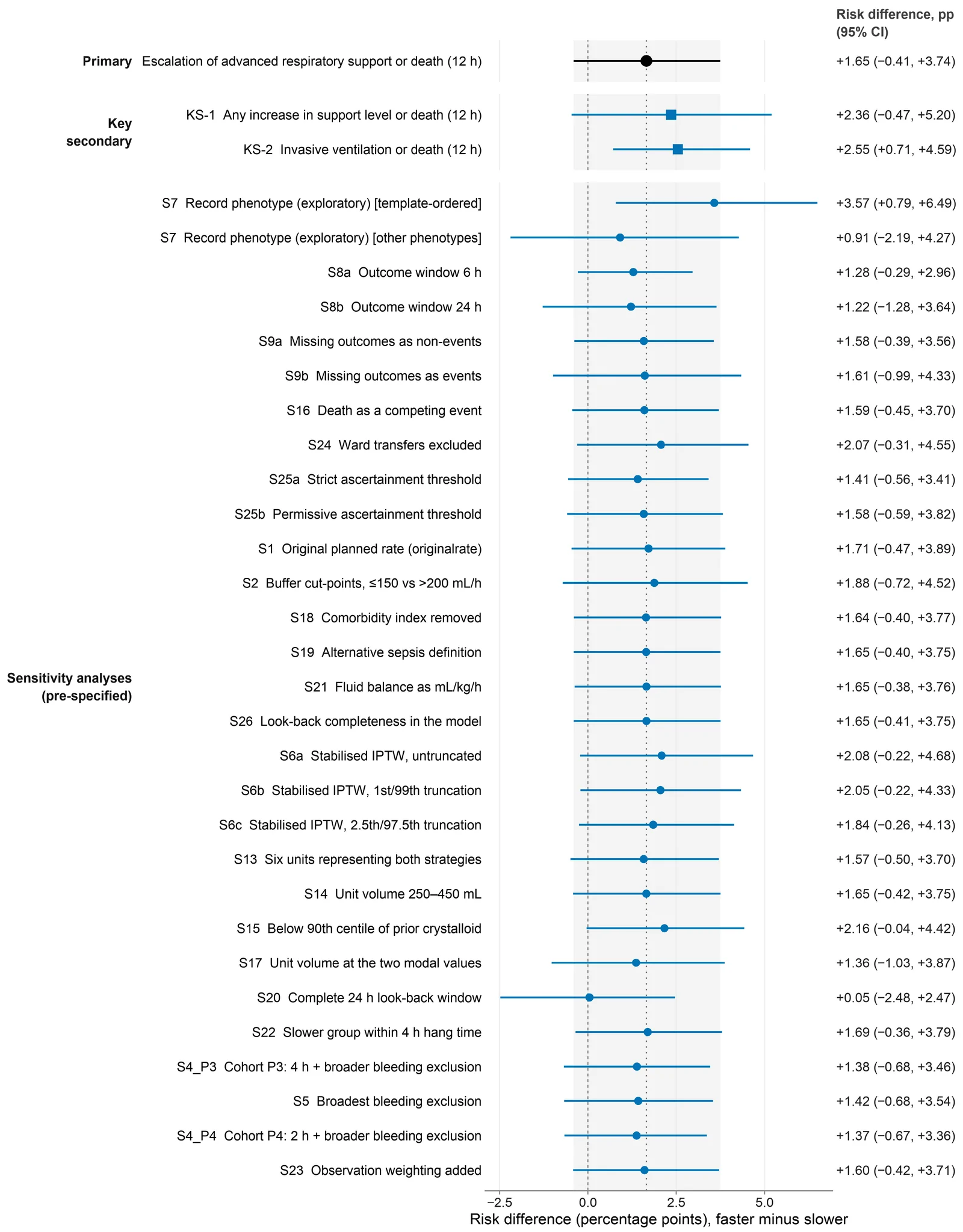
Risk differences for primary, key secondary, and pre-specified sensitivity analyses. Risk differences are expressed in percentage points and calculated as faster minus slower; positive values indicate a higher 12 h risk under the faster initial infusion-rate strategy. Points represent effect estimates and horizontal lines 95% confidence intervals. The dotted vertical line indicates the primary estimate, and the grey band is its 95% confidence interval. Confidence intervals were obtained from 2000 non-parametric bootstrap resamples with the propensity-score model refitted and overlap weights recomputed in each resample. Invasive mechanical ventilation or death is a nested key secondary outcome within the primary composite.

Among the 2671 analysed, 143 experienced the primary outcome: 88 of 1711 slower and 55 of 960 faster. Weighted 12 h risks were 4.95% and 6.60%, respectively. The marginal risk difference in the overlap population was 1.65 percentage points (95% CI −0.41 to 3.74), with a risk ratio of 1.33 (95% CI 0.93 to 1.92) (Table 3; Figure 3). The corresponding unweighted risks were 5.14% and 5.73%, a crude difference of 0.59 percentage points, so overlap weighting increased the magnitude of the contrast approximately threefold.

For the first key secondary outcome, any increase in respiratory support or death, the risk difference was 2.36 percentage points (95% CI −0.47 to 5.20). For invasive mechanical ventilation or death, weighted risks were 3.58% and 6.13%, corresponding to a risk difference of 2.55 percentage points (95% CI 0.71 to 4.59). This was the only primary or key secondary contrast whose 95% interval excluded no difference; no adjustment for multiplicity was applied. The two outcome definitions differed in event composition: among the 143 primary-outcome events, invasive mechanical ventilation accounted for 55/88 in the slower and 47/55 in the faster strategy, and escalation to advanced non-invasive support for 27/88 and 5/55 (Table 3). Death without qualifying respiratory support escalation accounted for the remaining 9/143 primary events (6.3%; six in the slower and three in the faster strategy). No other pre-specified secondary outcome had a confidence interval excluding no difference; 28-day mortality was −1.45 percentage points (95% CI −4.10 to 1.30) (Table S8).

### 3.4. Sensitivity Analyses and Subgroups

Across analyses estimating the faster-minus-slower risk difference for the primary outcome, and excluding the exploratory record-phenotype stratification, point estimates ranged from 0.05 to 2.16 percentage points and all 95% confidence intervals included no difference (Figure 3; Table S5). The pre-specified escalation-only analysis, which retained the primary advanced-support thresholds and treated death before qualifying escalation as a competing event, yielded a risk difference of 1.59 percentage points (95% CI −0.45 to 3.70) (S16). Most estimates were close to the primary result, although restriction to the 1504 patients with a complete 24 h covariate look-back window attenuated the risk difference to 0.05 percentage points (95% CI −2.48 to 2.47; 65 events) (S20); look-back completeness itself differed between strategies, at 53.33% in the slower and 61.71% in the faster. Excluding slower-strategy episodes exceeding four hours yielded 1.69 percentage points (95% CI −0.36 to 3.79) (S22), and excluding all 370 ward transfers left 2301 patients with the same 143 observed events and yielded 2.07 percentage points (95% CI −0.31 to 4.55) (S24). Alternative ascertainment thresholds are reported in Tables S4 and S5.

In the exploratory record-phenotype stratification, the risk difference was 3.57 percentage points (95% CI 0.79 to 6.49) in template-ordered records and 0.91 (−2.19 to 4.27) elsewhere; interaction was 2.66 (−1.89 to 7.04).

The pre-specified falsification analyses, applied to the 12 and 6 h periods before time zero in the 1548 and 1850 episodes with a determinable pseudo-baseline and a complete look-back, did not detect a difference between strategies (−1.57 percentage points, 95% CI −4.83 to 1.71, on 121 pre-window events, and −0.93, −3.19 to 1.21, on 90). The upper confidence limit of the 12 h analysis, 1.71 percentage points, is close to the primary estimate, so an imbalance of that magnitude before time zero is not excluded (Table S5). A negative-control outcome of new positive culture 72 h to 7 days after time zero gave 0.85 percentage points (−1.71 to 3.29).

The E-value was 2.00 for the primary risk ratio and 1.00 for the confidence limit closest to the null; corresponding values for invasive mechanical ventilation or death were 2.81 and 1.60.

Four of five pre-specified subgroups were restricted to descriptive reporting because of the event-count gate; the strategy-representation gate was met in all strata (Figure S3; Supplementary Methods S7). Only the sepsis subgroup met both gates for quantitative estimation. Without adjustment for multiplicity, risk differences were 0.57 percentage points (95% CI −2.80 to 4.16) with sepsis and 2.65 percentage points (95% CI 0.13 to 5.22) without sepsis; the interaction was −2.08 percentage points (95% CI −6.42 to 2.37). The interaction estimate was imprecise and did not distinguish the strata.

### 3.5. Dose–Response

**Figure 4:**
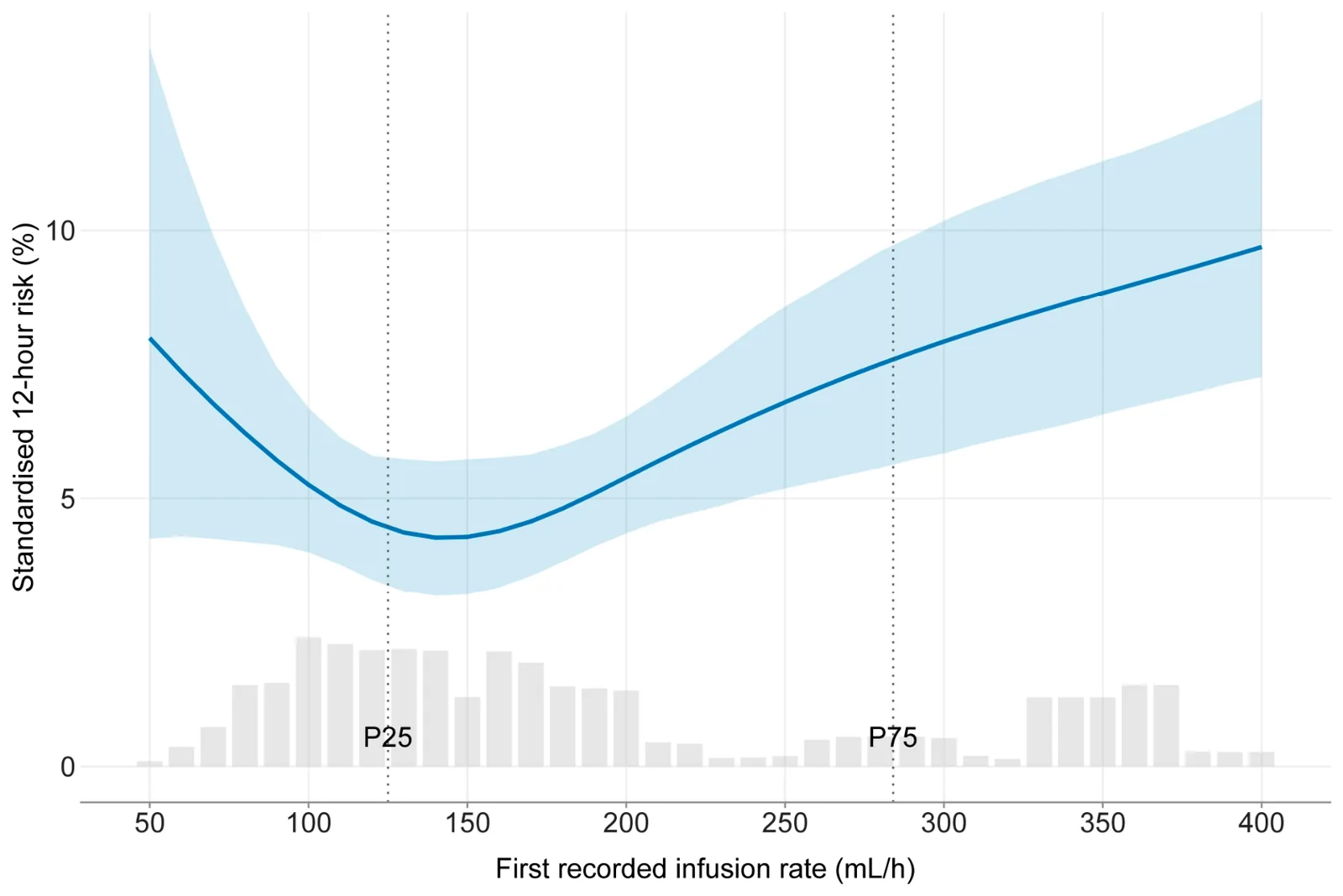
Dose–response relation between initial infusion rate and 12 h risk. Standardised 12 h risk of escalation of advanced respiratory support or death, by initial infusion rate, obtained by g-computation over the pooled ascertainable cohort of 2882 episodes with 178 events, including 211 of the 226 episodes above 350 mL/h. Shading is the 95% percentile bootstrap band from 2000 resamples.

The pre-specified dose–response analysis used g-computation over the pooled ascertainable cohort of 2882 episodes with 178 events, including 211 of the 226 episodes above 350 mL/h, and does not share the estimand of the primary comparison (Figure 4). Standardised 12 h risk was lowest at about 140 mL/h (4.27%, 95% CI 3.18 to 5.68) and rose as the rate increased above that point, to 6.79% (5.18 to 8.56) at 250 mL/h and 8.83% (6.56 to 11.28) at 350 mL/h. Below 140 mL/h, the fitted risk also rose, reaching 7.98% (95% CI 4.25 to 13.38) at 50 mL/h; data density was low, with 36 episodes within 25 mL/h of 50 mL/h. The contrast between the 75th and 25th centiles of rate was 3.13 percentage points (95% CI 0.47 to 5.80); restricted to the cohort below 350 mL/h and evaluated at the same rates, it was 1.33 percentage points.

## 4. Discussion

In this target trial emulation of 2790 critically ill adults receiving a first red blood cell (RBC) transfusion, the primary analysis included 2671 patients and 143 events. The estimated risk difference comparing the faster with the slower strategy for escalation to advanced respiratory support or death within 12 h was 1.65 percentage points (95% CI −0.41 to 3.74). This interval encompassed both no difference and a potentially clinically important increase in risk; the result therefore neither demonstrates equivalence nor supports changing practice. Excluding the exploratory record-phenotype stratification, sensitivity estimates ranged from 0.05 to 2.16 percentage points and all corresponding 95% CIs included no difference. The pre-specified outcome of invasive mechanical ventilation or death, nested within the primary composite, yielded a risk difference of 2.55 percentage points (95% CI 0.71 to 4.59).

Most clinical evidence on RBC transfusion has focused on thresholds and transfused volume rather than administration rate [30,31,32]. Evidence specifically addressing rate in adults remains sparse and has evaluated different outcomes. In a prospective medical ICU study, patients who developed transfusion-associated circulatory overload (TACO) received blood components faster than matched controls, although rate was assessed alongside transfusion volume and fluid balance rather than as a predefined initial RBC strategy [9]. A hospital-wide analysis of 2359 RBC infusions found no clear association between rate and clinically significant vital-sign changes but did not evaluate respiratory support escalation [33]. A randomised rat model supports physiological plausibility, showing that faster RBC infusion increased left ventricular end-diastolic pressure, particularly at higher transfusion volumes [6]. Our outcome captures a clinically consequential change in respiratory support rather than adjudicated TACO, which can be difficult to distinguish from other pulmonary deterioration in critical illness [10,23,24,25]. This study therefore extends the literature by evaluating predefined initial-rate strategies against a respiratory outcome that is observable independently of whether TACO is formally adjudicated.

If the haemodynamic mechanism suggested by the experimental evidence above operates in this population, its effect would be expected to concentrate in the most severe component of a composite outcome and to be more apparent at higher rates—a pattern broadly consistent with, though not established by, the present findings. The key secondary and dose–response results accordingly require cautious interpretation. Because invasive mechanical ventilation or death was nested within the primary composite, it is not an independent replication of the primary comparison. The post hoc decomposition likewise cannot establish that faster infusion selectively increases severe events while reducing non-invasive escalation, because the components compete and are constrained to sum to the composite. The pre-specified exploratory dose–response analysis showed a non-monotone fitted association, with the lowest standardised risk near 140 mL/h and higher fitted risks at the upper end of the rate distribution. However, it used a different estimand, included rates above 350 mL/h, and had sparse data at the lower end. The fitted minimum therefore does not identify an optimal rate or causal threshold; it defines a hypothesis for prospective evaluation.

These findings do not establish the safety of either strategy or justify selecting one rate range over the other. They nevertheless provide estimates of eligibility, rate separation, event frequency, and uncertainty that may inform feasibility and scenario-based sample-size planning for a prospective multicentre study; its target effect should be chosen independently on clinical grounds. Because haemodynamic conditions may evolve during transfusion, such a study could assign an initial-rate protocol while allowing pre-specified safety-driven adjustments, incorporating delivery-verified exposure and complete post-transfer outcome ascertainment to address the principal limitations detailed below. The post hoc process audit indicated that four-hour completion depended jointly on recorded unit volume, starting rate, and subsequent adjustments; without removal-from-storage times or pump-derived completion times, these observations describe documented workflow rather than verified compliance and do not support a universal starting-rate threshold [7,11].

Strengths include explicit specification of eligibility, strategies, time zero, follow-up, and outcomes within a target-trial framework. Using the recorded starting rate to classify strategy at the start of follow-up avoided assigning exposure using completion-dependent information. The protocol and analysis plan preceded exposure–outcome modelling; pre-specified analyses were reported regardless of their results, and exploratory and post hoc analyses were labelled. Overlap weighting balanced measured baseline covariates, each bootstrap resample refitted the propensity-score model, and respiratory support was ascertained using a pre-specified algorithm integrating multiple record sources. These features improve design transparency but cannot substitute for random assignment.

Several limitations remain. First, rate was clinically selected rather than randomised. Overlap weighting cannot address unrecorded determinants of rate and outcome, including bedside assessments of haemodynamic instability, bleeding, and volume tolerance; RBC storage duration and other unavailable component characteristics also could not be adjusted for. Conditional exchangeability cannot be verified, and residual bias may operate in either direction. Second, exposure was the first documented setting rather than pump-derived delivered flow [34], and multiple record segments were more frequent under the slower strategy. Actual delivered flow and changes across strategy ranges were not verified or incorporated, so the results concern the initial strategy rather than a sustained rate. The concurrent-transfusion proxy counted records in a 15 min window after initiation and could therefore exclude segmented records from one episode or introduce selection based on post-time-zero information; episode identifiers do not independently verify physical blood units. Third, baseline data came from variable-length look-back periods and some comorbidities were hospitalisation-code-derived proxies; restricting analysis to a complete 24 h history attenuated the estimate but substantially reduced the event count. Fourth, the outcome reflected clinician-initiated respiratory support rather than an adjudicated cause and may vary with local practice. Support after ward transfer was unavailable, requiring no escalation to be assumed for 13.3% of included patients; differential misclassification remains possible. Excluding these patients produced an imprecise estimate but changed the analysed population and could itself introduce selection bias; absence of ICU readmission did not verify absence of ward escalation. Finally, this single-centre study covered 2008–2022 and was restricted to first ICU transfusions, rates of 50–350 mL/h, patients without high-specificity bleeding markers, and the empirical overlap population. The longer median interval from ICU admission to first RBC recording (28.98 versus 9.75 h; Table S10) partly reflects the eligibility requirement of at least four hours between ICU admission and transfusion initiation, which excluded 3902 recipients. These comparisons describe the selection of the study population rather than establishing transportability of the weighted association. Generalisation beyond these settings should be cautious.

## 5. Conclusions

Among critically ill adults receiving a first red cell transfusion, a faster versus slower initial infusion rate was not associated with a statistically distinguishable difference in escalation to advanced respiratory support or death within 12 h. However, the confidence interval did not exclude a clinically important increase in risk, and the nested secondary outcome of invasive mechanical ventilation or death showed a signal of potential harm with faster infusion. These findings neither justify a change in current infusion-rate practice nor support the assumption that infusion rate is clinically inconsequential. Rather, they identify infusion rate as a potentially modifiable yet insufficiently studied component of transfusion delivery and provide empirically grounded estimates of the relevant population, exposure contrast, event frequency, and effect size needed to inform the design of prospective studies capable of determining whether infusion rate itself influences early respiratory deterioration.

## Figures and Tables

**Table 1:** The target trial and its emulation.

Element	Target Trial	Emulation in This Study
Eligibility	Adults in intensive care at their first red cell transfusion, with a first recorded infusion rate of 50–350 mL/h, at least 4 h after admission, without high-specificity markers of bleeding or a bleeding-prone context, and with an ascertainable baseline respiratory level below invasive ventilation.	Same as target trial
Strategies	Slower: infuse at 50–175 mL/h. Faster: infuse at >175–350 mL/h. The assigned initial rate is set at transfusion initiation; subsequent clinically indicated rate adjustments are permitted and do not alter assignment to the initial-rate strategy.	Assigned by the rate recorded on the first line of the first episode, under the assumption that this reflects the strategy implemented at time zero. A later change in rate does not alter the classification; later rate, hang time and completion take no part in assignment and are reported as implementation after time zero
Assignment	Random, unblinded	No randomisation. Overlap weighting adjusted measured covariates, without verified exchangeability. The initial recorded rate was not protocol-assigned; intention-to-treat or per-protocol effects are not claimed.
Time zero	Randomisation	First record of the first RBC episode. Initial strategy and follow-up start together, without a grace period.
Follow-up	12 h from time zero	Same as target trial
Outcome	Escalation of advanced respiratory support or death within 12 h	Same as target trial
Causal contrast	Intention-to-treat effect	Weighted marginal absolute risk difference, faster minus slower, in the overlap population
Analysis	Compare risks between assigned groups	Overlap weighting; 2000 non-parametric bootstrap resamples with propensity scores and weights re-estimated in each resample; pre-specified sensitivity and falsification analyses; E-values. Post hoc analyses were explicitly labelled.

**Table 2:** Baseline characteristics of the analysed cohort.

Variable	Slower, Unweighted	Faster, Unweighted	SMD, Unweighted	Slower, Overlap Weighted	Faster, Overlap Weighted	SMD, Overlap Weighted
— Exposure, fixed at time zero —						
First recorded infusion rate (mL/h)	127.5 (31.6)	286.0 (66.7)	—	132.6 (32.1)	277.1 (68.4)	—
— Baseline covariate: the unit —						
Recorded starting unit volume (mL)	338.0 (37.5)	339.5 (35.6)	0.041	339.7 (35.8)	339.7 (36.9)	0.000
— Demographics and admission —						
Age (years)	68.1 (15.3)	66.1 (14.2)	−0.139	66.4 (14.8)	66.4 (14.7)	−0.000
Female	48.8%	44.8%	−0.081	46.1%	46.1%	−0.000
Weight (kg)	79.2 (22.0)	79.6 (20.0)	0.021	79.6 (20.9)	79.6 (20.6)	0.000
Hours from intensive care admission to time zero	46.0 (67.1)	43.9 (59.3)	−0.034	44.5 (62.0)	44.5 (66.8)	−0.000
First intensive care stay	76.2%	78.7%	0.060	77.2%	77.2%	0.000
Started at night	29.2%	28.6%	−0.013	29.6%	29.6%	0.000
Started at a weekend	29.9%	26.9%	−0.067	28.4%	28.4%	0.000
— Severity of illness —						
SOFA score at time zero	4.12 (2.72)	4.07 (2.83)	−0.018	4.10 (2.86)	4.10 (2.86)	0.000
Sepsis at time zero	51.9%	47.1%	−0.096	48.0%	48.0%	0.000
Haemoglobin (g/dL)	7.46 (1.08)	7.73 (1.20)	0.241	7.62 (1.17)	7.62 (1.18)	−0.000
Noradrenaline equivalent (µg/kg/min)	0.02 (0.08)	0.02 (0.08)	0.025	0.02 (0.08)	0.02 (0.08)	−0.000
Charlson comorbidity index	6.19 (3.02)	5.46 (2.83)	−0.249	5.71 (2.90)	5.71 (2.92)	−0.000
Heart failure	40.0%	25.6%	−0.309	29.9%	29.9%	0.000
Chronic kidney disease or end-stage renal disease	32.1%	26.5%	−0.122	27.8%	27.8%	0.000
Receiving renal replacement therapy	5.9%	6.6%	0.029	6.8%	6.8%	0.000
— Volume status —						
24 h net fluid balance (mL)	4149.8 (9532.2)	3531.5 (7358.7)	−0.073	3914.5 (8904.0)	3914.5 (8423.7)	−0.000
Crystalloid in the 6 h before time zero (mL/kg)	4.17 (8.59)	5.91 (43.37)	0.055	4.61 (9.77)	5.94 (43.44)	0.042
Colloid in the 6 h before time zero (mL/kg)	0.17 (1.13)	0.66 (2.29)	0.270	0.34 (1.56)	0.34 (1.62)	0.002
Loop diuretic before time zero	31.6%	35.5%	0.082	31.9%	31.9%	0.000
— Baseline respiratory support level —						
0 Room air or no oxygen	33.8%	29.3%	−0.097	31.6%	31.6%	−0.000
1 Conventional oxygen	61.7%	66.1%	0.091	63.9%	63.9%	0.000
2 High-flow nasal cannula	2.8%	3.0%	0.012	2.8%	2.8%	0.000
3 Non-invasive ventilation	1.7%	1.6%	−0.007	1.7%	1.7%	0.000
— Intensive care unit type (13 categories; full breakdown in Table S6) —						
The largest unweighted standardised difference in the table, 0.634, is in this block, at Cardiac Vascular Intensive Care Unit (CVICU); it is 0.000 after weighting.						
— Admission type —						
Elective	12.4%	24.4%	0.315	18.7%	18.7%	0.000
Emergency	74.0%	62.4%	−0.250	67.6%	67.6%	−0.000
Observation	13.7%	13.2%	−0.014	13.7%	13.7%	0.000
— Post-time-zero infusion process (not baseline covariates; not balanced) —						
Whole-episode hang time (min)	157.8 (42.9)	75.9 (23.6)	—	151.7 (42.2)	78.7 (24.9)	—
— Descriptive variables outside the propensity score model —						
Lactate measured	32.6%	31.8%	−0.016	31.8%	32.1%	0.006
Complete 24 h look-back window	53.3%	61.7%	0.170	56.7%	57.1%	0.006

**Table 3:** Outcomes.

Outcome	Events/*n*, Slower	Events/*n*, Faster	Weighted Risk, Slower	Weighted Risk, Faster	Risk Difference, Percentage Points (95% CI)	Risk Ratio (95% CI)
Primary: escalation of advanced respiratory support or death (12 h)	88/1711	55/960	4.95%	6.60%	+1.65 (−0.41, +3.74)	1.33 (0.93, 1.92)
Key secondary 1: any increase in respiratory support level or death (12 h)	180/1711	116/960	10.57%	12.93%	+2.36 (−0.47, +5.20)	1.22 (0.96, 1.55)
Key secondary 2: invasive mechanical ventilation or death (12 h)	61/1711	50/960	3.58%	6.13%	+2.55 (+0.71, +4.59)	1.71 (1.16, 2.58)

## Data Availability

The locked protocol, statistical analysis plan, analysis code, run log and derived non-identifiable results are openly available at the Open Science Framework: https://osf.io/cmeys/ (DOI 10.17605/OSF.IO/CMEYS). MIMIC-IV v3.1 is available through PhysioNet (https://physionet.org/content/mimiciv/3.1/) to credentialed researchers who complete the required training and sign the data use agreement. Access to patient-level data is governed by the PhysioNet Data Use Agreement. The datasets used and/or analysed during the current study are available from the corresponding author on reasonable request.

## Supplementary Materials

The following supporting information can be downloaded at https://www.mdpi.com/article/10.3390/jcm15208147/s1, Supplementary Methods S1. Exposure definition; Supplementary Methods S2. Respiratory-support ladder and outcome ascertainment; Supplementary Methods S3. Covariates; Supplementary Methods S4. Weighting, balance and inference; Supplementary Methods S5. Pre-specified sensitivity analyses; Supplementary Methods S6. Post-hoc process audit of unit completion; Supplementary Methods S7. Pre-specified subgroups and the gating rule; Supplementary Methods S8. Software and reproducibility; Supplementary Methods S9. Post hoc descriptive population comparison; Figure S1: Covariate balance; Figure S2: Propensity-score overlap; Figure S3: Subgroup representation and the gating rule; Figure S4: Distributional balance; Figure S5: Whether the starting rate could complete the unit within four hours; Table S1: Respiratory-support level mapping; Table S2: Excluded and unascertained patients; Table S3: Outcome ascertainability; Table S4: Alternative ascertainment thresholds; Table S5: Sensitivity analyses and E-values; Table S6: Full cohort characteristics; Table S7: Transitions for the first key secondary outcome; Table S8: Other secondary outcomes; Table S9: Outcome validity by level of support reached; Table S10A: Included and non-included ICU RBC recipients; Table S10B: Sequential exclusions from 15,045 ICU RBC recipients. The TARGET reporting checklist is submitted as a separate file.

## Abbreviations

CI	Confidence Interval
HFNC	High-Flow Nasal Cannula
IMV	Invasive Mechanical Ventilation
IQR	Interquartile Range
NIV	Non-Invasive Ventilation
OW	Overlap Weighting
RBC	Red Blood Cell
RD	Risk Difference
RR	Risk Ratio
SMD	Standardised Mean Difference
SOFA	Sequential Organ Failure Assessment
TACO	Transfusion-Associated Circulatory Overload
