# Supplementary material for: Initial Red Cell Infusion Rate and Escalation of Respiratory Support in Critically Ill Adults: A Target Trial Emulation
Source: J Clin Med. 2026 Oct 20;15(20):8147. doi: 10.3390/jcm15208147 (PMC13705826; doi:10.3390/jcm15208147)
Supplement: Supplementary file 1 [file jcm-15-08147-s001.zip › jcm-4574516-supplementary.pdf]

Supplementary Methods

S1. Exposure definition

Treatment strategy was classified from the rate recorded at initiation of the first eligible RBC transfusion episode (inputevents.rate). The first recorded rate determined classification as slower (50–175 mL/h) or faster (>175–350 mL/h). Subsequent rate changes, whole-episode hang time, delivered volume, and transfusion completion did not alter this classification. This definition aligns treatment classification with time zero and avoids using information arising after follow-up has begun to define baseline exposure. It relies on the measurement assumption that the first recorded rate adequately represents the initial rate implemented in clinical care. Subsequent rate changes were treated as post-time-zero implementation characteristics and were described but not used to redefine treatment strategy. Multiple infusion-record segments were counted descriptively as an implementation characteristic. Such segmentation can reflect changed recorded rates or interruptions and does not establish pump-measured delivery or crossover between strategy ranges. In S1, patients were regrouped using originalrate, the care-provider-entered original planned rate in the same first inputevents record, applying the same cut-points to values of 50–350 mL/h and refitting the propensity-score model.

The rate achieved over the entire transfusion was not used as an alternative exposure because it becomes known only after follow-up has begun and may incorporate subsequent clinical decisions and responses to the patient's evolving condition. A sustained-rate strategy requiring maintenance of a specified rate until completion would represent a different treatment strategy and estimand and was not the target of the present emulation. We therefore refer throughout to initial-rate strategies rather than to intention-to-treat or per-protocol effects.

Recorded starting unit volume was defined as originalamount on the first infusion record. Within transfusion episodes, this field behaved as the volume remaining at the beginning of each recorded segment: 2,839 of 2,865 between-segment transitions (99.09%) satisfied originalamount(next) = originalamount(current) − amount(current). Accordingly, the first-record value was interpreted as the volume present when the recorded infusion episode began and was treated as a baseline characteristic available at time zero. It was not interpreted as prescribed volume or total volume ultimately delivered, because units stopped before completion could have a recorded starting volume exceeding the volume actually transfused. The terms recorded starting unit volume and rate recorded at initiation are used throughout.

S2. Respiratory-support ladder and outcome ascertainment

Support was represented on five levels: room air or no oxygen (0); conventional oxygen, including tracheostomy mask without mechanical ventilation (1); high-flow nasal cannula or non-invasive ventilation (2–3, combined as a single non-invasive advanced tier because both occupy the same position in nursing escalation decisions); and invasive mechanical ventilation (4). The pre-specified escalation-only analysis (S16) used the primary outcome's advanced-support thresholds, treating death before qualifying escalation as a competing event. In this fixed 12-hour binary risk analysis, patients who died without qualifying escalation remained in the denominator without a qualifying escalation event (Table S5).

Persistence thresholds differed by level: invasive ventilation required a single record; the non-invasive tier required an interval overlapping the window by ≥30 minutes or two point records ≥15 minutes apart, which excludes transient device trials, nebulisation and transport artefacts; conventional oxygen and room air required a single record.

Two record types were treated as positive evidence of no escalation rather than as missing: device text recording no oxygen, and the supplemental-oxygen state in the derived ventilation table.

Respiratory negative-control outcomes are reported using two criteria. The pre-specified criterion counts any increase in support level. The construct-matched criterion uses the advanced-support thresholds of the primary outcome and is labelled post hoc. Estimates are in Table S5.

The outcome records a clinician-initiated change in delivered support, not a diagnosis. Protocol §5.2 forbids reading it as transfusion-associated circulatory overload, which cannot be reliably separated in intensive care from other transfusion-related respiratory complications [24].

Ascertainment. Each patient falls in one of six mutually exclusive categories (Table S3), assigned using a time-ordered rule that prioritises death and observed escalation over ward transfer and truncated observation. Patients with unclassifiable respiratory records or truncated observation were excluded from the analysed population; ward transfers without an observed event were included as assumed non-events.

The ward-transfer assumption. The two components of the composite are not observed over the same territory. Death is taken from the hospital-wide admission record, so a ward death within 12 hours is captured. Respiratory support is assembled from four intensive care sources, so once a patient leaves, no record exists. For the 370 patients (13.26% of the included 2,790, or 13.85% of the classified 2,671) discharged to a general ward before 12 hours, non-escalation is assumed, and the assumption can fail in one direction only — a ward escalation recorded as a non-event. Discharge occurred at a median of 7.35 hours after time zero (IQR 4.69–10.02); none re-entered intensive care within the window, 126 left within six hours and 46 within three; none died within the window. Ward transfer was more frequent under the faster strategy (14.86% versus 12.37%), so classification may be differentially affected by unobserved ward escalation; these transfer counts do not establish the presence, magnitude or direction of bias in the strategy contrast. Excluding all 370 (S24) leaves the event counts unchanged, since only non-events are removed, and moves the estimate from 1.65 to 2.07 percentage points (95% CI −0.31 to 4.55), with 2,301 patients and 143 events. This is a restriction to a different population, not recovery of unobserved ward respiratory support or proof that informative transfer is harmless.

Strict and permissive threshold variants change both whether an event is counted and whether the outcome is ascertainable, so the analysis population differs between variants (2,654 / 2,671 / 2,671). Event counts are in Table S4 and the corresponding estimates, in variant-specific and fixed-N versions, in Table S5.

S3. Covariates

Pre-specified: age, sex, weight, unit type, admission type, admission year group, hours from

admission to time zero, first stay, SOFA at time zero, sepsis at time zero, haemoglobin, noradrenaline equivalent dose and its 2-hour trend, Charlson index, heart failure, chronic kidney disease or end-stage renal disease, active renal replacement therapy, 24-hour net fluid balance, crystalloid and colloid volume per kilogram over the preceding 6 hours (log1p), prior loop diuretic, baseline respiratory level, recorded starting unit volume, and two workflow timing indicators (night start, weekend start). Continuous covariates entered as restricted cubic splines with knots at the 5th, 35th, 65th and 95th percentiles; degenerate knots reduced the term to linear automatically, with the reduction logged.

RBC storage duration was unavailable in the structured transfusion records and was not adjusted for. Documentation/validation time (storetime) does not represent component storage age; recorded infusion duration and removal-from-storage time likewise do not measure it.

All windowed covariates were computed over [max(admission, time zero − window), time zero). SOFA was the last score ending at or before time zero, and sepsis required a suspected-infection time at or before time zero together with that score. These timing restrictions apply to time-stamped clinical variables. The Charlson index, heart failure and chronic kidney disease were derived from hospitalisation diagnoses, which do not verify whether the condition was present or known at time zero. They were treated as hospitalisation-code-derived proxies rather than verified time-zero diagnoses.

Missing data. A three-tier rule was fixed before missingness was measured: below 5%, median or mode imputation plus a missing indicator; 5–20%, retain the indicator and state the limitation; above 20%, remove from the main model. All covariates but three were complete. Haemoglobin (2.29%) and SOFA (0.04%) fell in the first tier; lactate (67.7%) in the third and was removed. No covariate fell in the middle band. The locked implementation uses mean rather than median imputation; a median variant was run in parallel and gave identical propensity-score diagnostics. No patient was dropped for a missing covariate, so the analysis is not complete-case. Multiple imputation (S11) was not performed because all retained model covariates other than haemoglobin had less than 1% missingness, meeting the pre-specified criterion for omitting this analysis.

Excluded by design. The record phenotype is a function of the exposure and was used only descriptively and in one exploratory stratification. Look-back completeness is near-collinear with the spline for hours from admission and entered only in a sensitivity analysis.

S4. Weighting, balance and inference

For the primary analysis, propensity scores were estimated by logistic regression fitted by iteratively reweighted least squares in the full cohort of 2,790. The resulting overlap weights were applied to patients with ascertainable outcomes, without refitting the model solely because of restriction by outcome ascertainability. Exact mean balance is therefore a property of the 2,790, holding approximately in the 2,671. Overlap weights are bounded by construction and were not truncated; truncation at the 1st/99th and 2.5th/97.5th percentiles applied only to the stabilised inverse-probability weights used as a sensitivity analysis. Sensitivity analyses that redefined exposure or model specification refitted the propensity-score model.

Balance was assessed by standardised mean differences without P values, and additionally by

Kolmogorov–Smirnov distances, variance ratios against the Rubin range of 0.5–2.0, and standardised differences after clinical binning (Figures S1, S4). Because exact mean balance of in-model covariates is a mathematical property of overlap weighting, the informative checks are those on variables outside the model and on three pre-specified interactions.

Bootstrap. 2,000 non-parametric resamples of intensive care stays, seed 20260803, percentile intervals. Every resample repeated the whole chain — resample, refit the propensity-score model, recompute weights, restrict to the analysis population, estimate — because resampling only the final estimator would exclude the uncertainty of propensity-score estimation and give intervals that are systematically too narrow. Resamples missing a care-unit category dropped that indicator column; this was pre-specified, arises from six units contributing fewer than 25 patients each, and is not counted as a failure. Non-convergent resamples were recorded as failures and replaced; failures are reported for every analysis.

S5. Pre-specified sensitivity analyses

All twenty-six were specified in the locked protocol before the cohort was built. Twenty-five were run; S11, multiple imputation, was not performed under the pre-specified missing-data rule described in Section S3. Each analysis appears as the correspondingly numbered row of Table S5, with three exceptions: S3, the continuous-rate spline, is reported as the dose–response analysis in Figure 4; S10 is the E-value block of Table S5; and S12, the negative controls, occupies rows S12a–S12c. The unit-completion audit and the fixed-N variants of S25 are post hoc and lie outside the twenty-six.

Bleeding exclusion tiers. The main cohort excludes six high-specificity markers. The broader tier adds patients meeting at least two of four supportive signals (haemoglobin range ≥2 g/dL over 24 hours; chest and surgical drainage above the 95th centile; crystalloid plus colloid above the 95th centile; rising noradrenaline requirement over the preceding 2 hours). The broadest tier adds admission-level bleeding diagnoses, excluded from the main definition because the diagnosis file carries no present-on-admission flag and bleeding arising during admission may lie downstream of the exposure.

S6. Post-hoc process audit of unit completion

Devised after the analyses; not part of the locked protocol.

The four-hour maximum applies to a unit, and the rate a unit needs depends on how much is in it, so no fixed rate can serve as a threshold for every unit: 350 mL requires 87.5 mL/h, 375 mL requires 93.75 mL/h. Each starting rate was therefore compared with that unit's own recorded starting volume divided by four hours.

In the slower group 123 of 1,787 units (6.9%) began below their own minimum. In the faster group none did, but this is forced by the strategy definition: the largest starting volume was 500 mL, whose four-hour minimum of 125 mL/h lies below the 175 mL/h floor of the faster strategy. The between-arm comparison therefore carries no information; only the 123 do. Completion is taken from the terminal charted status of the infusion rather than inferred from the interval.

Outcome	n	With a documented upward rate adjustment

Hung beyond four hours	48	4
Whole unit delivered within four hours (charted FinishedRunning)	59	59
Ended within four hours with the unit unfinished (Stopped or Paused)	16	8

Those 48 account for 48 of the 52 episodes with recorded hang time exceeding four hours; the other 4 began at or above their own minimum and were interrupted. The 16 comprise 14 Stopped and 2 Paused, with 29.6% to 93.7% of the unit given; they met the four-hour interval only because the infusion ended before the unit finished. A volume-based completion rule agrees in 74 of the 75 units ending within four hours, differing in one charted as FinishedRunning with 350 of a recorded 375 mL given; under that rule the second and third rows read 58 and 17.

Counts are reported rather than any measure of discriminative performance: hang time is close to a deterministic function of volume and rate, so such a measure would largely restate that arithmetic. The informative content is the departure from it — units rescued by an upward adjustment, units ended before completion, and episodes with recorded hang time exceeding four hours despite an adequate starting rate.

S7. Pre-specified subgroups and the gating rule

Five subgroups were pre-specified, all exploratory: heart failure; age ≥75 years; chronic kidney disease; positive fluid balance at baseline; sepsis at time zero. No multiplicity adjustment is applied and none is confirmatory.

Two gates were fixed in the protocol and applied before any effect estimate was inspected: a stratum was restricted to descriptive reporting if either rate group fell below 5% of that stratum, or if either arm contained fewer than 25 events. The order matters — the gates are a property of the stratum, not of the estimate, so applying them after inspection would convert them into a selection rule on results.

The representation gate was met everywhere; the event-count gate was binding in four of five subgroups, leaving sepsis as the only stratum with an estimate (Figure S3). This is a statement about precision: four down-graded subgroups mean the study is underpowered to examine effect modification, and this must not be read as an absence of it.

S8. Software and reproducibility

Cohort construction and outcome derivation in PostgreSQL 16; analysis in Python 3 with NumPy; figures in R with ggplot2. Every reported value is traceable to a named script, its SHA-256 hash, its execution time and its output file, recorded in the run log. The cohort fingerprint df49857b312b7cf7f159948140418982 is asserted by 14 hard gate checks re-run after every change to the build pipeline. Protocol, code, run log and results are registered at https://osf.io/cmeys/ (DOI 10.17605/OSF.IO/CMEYS).

S9. Post hoc descriptive population comparison

Table S10 presents a post hoc descriptive comparison of included and non-included ICU RBC recipients. Each source-population patient contributed the first ICU stay in which an RBC

record occurred, as in the cohort-selection scheme. The study cohort comprised 2,790 included patients; all other 12,255 recipients were classified as not included. Demographic and admission descriptors used index ICU admission as a common anchor, including age calculated as anchor_age + calendar year of ICU admission − anchor_year. Time to first RBC used the earliest recorded RBC start in that stay. Continuous variables are summarised by median and interquartile range and categorical variables by number and percentage; missingness is reported, with no imputation, hypothesis testing, outcome comparison or reweighting. Sequential exclusion counts follow the study eligibility criteria.

Supplementary Figures

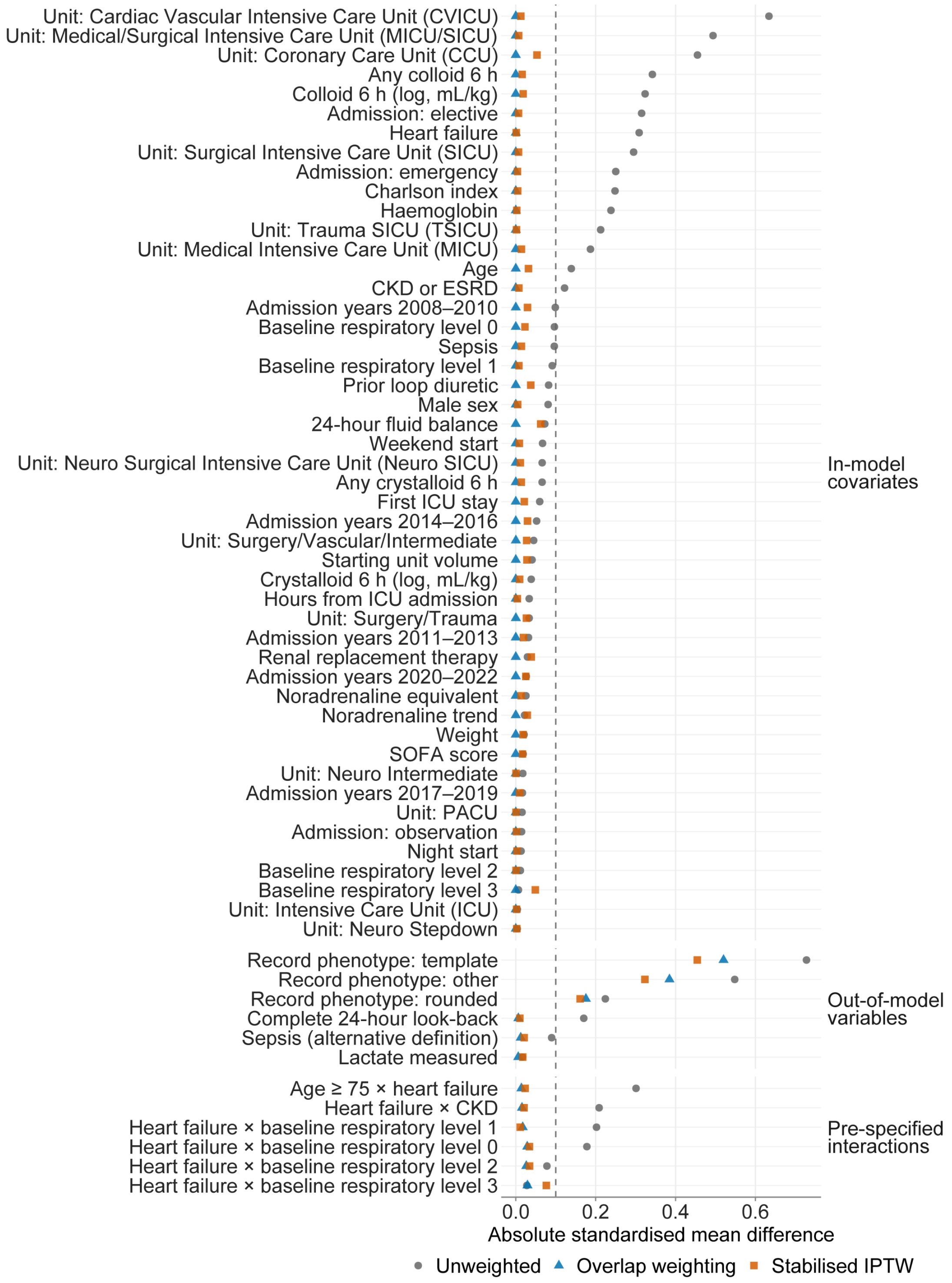

Figure S1. Covariate balance.

Standardised mean differences before and after overlap weighting, for covariates entered in the propensity-score model, for variables outside the model, and for three pre-specified interaction terms.

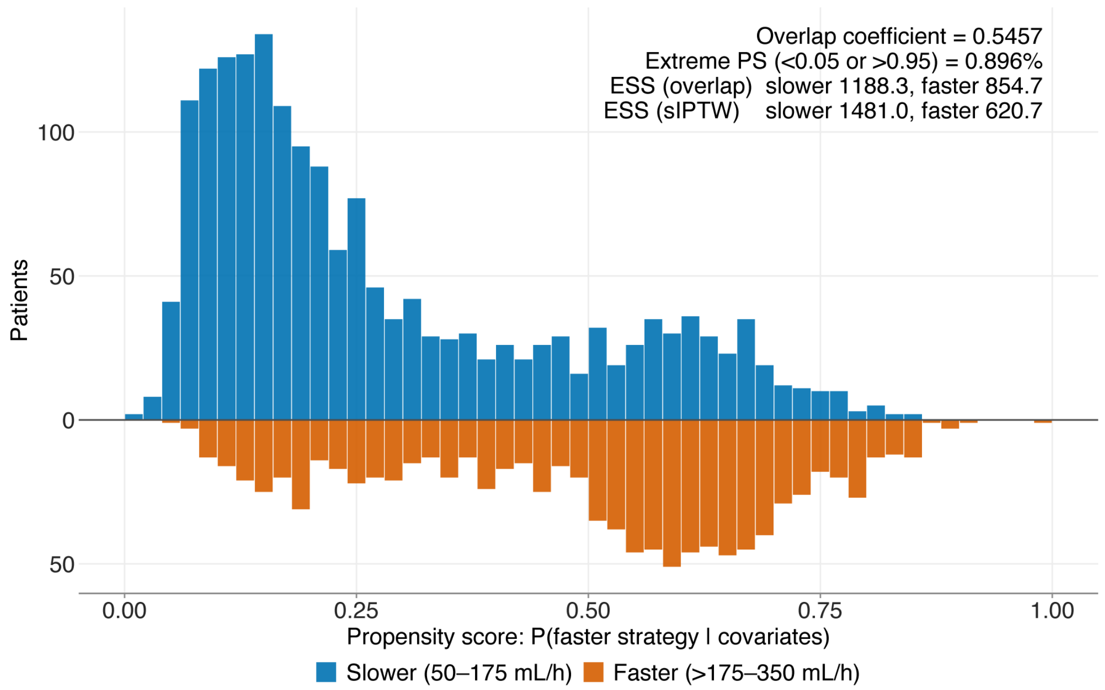

Figure S2. Propensity-score overlap.

Distribution of the estimated propensity score under each strategy.

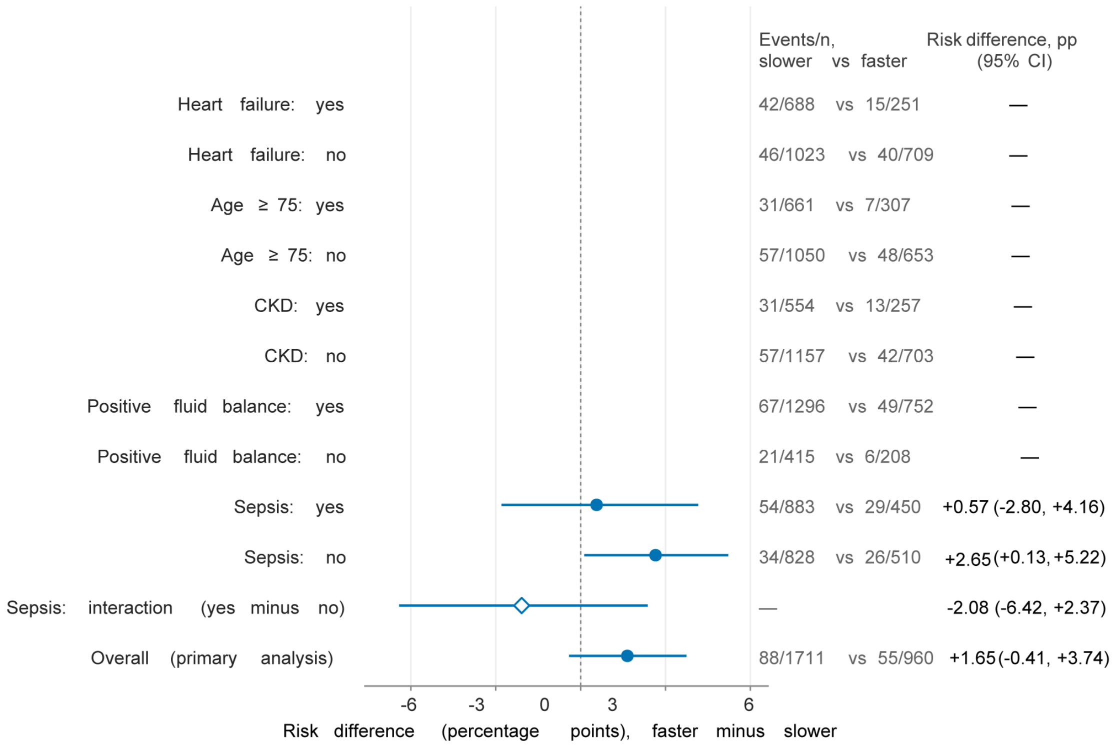

Figure S3. Subgroup representation and the gating rule.

For each of the five pre-specified exploratory subgroups, the representation of both strategies and the event counts, against the two gates fixed in the protocol and applied before any

estimate was inspected.

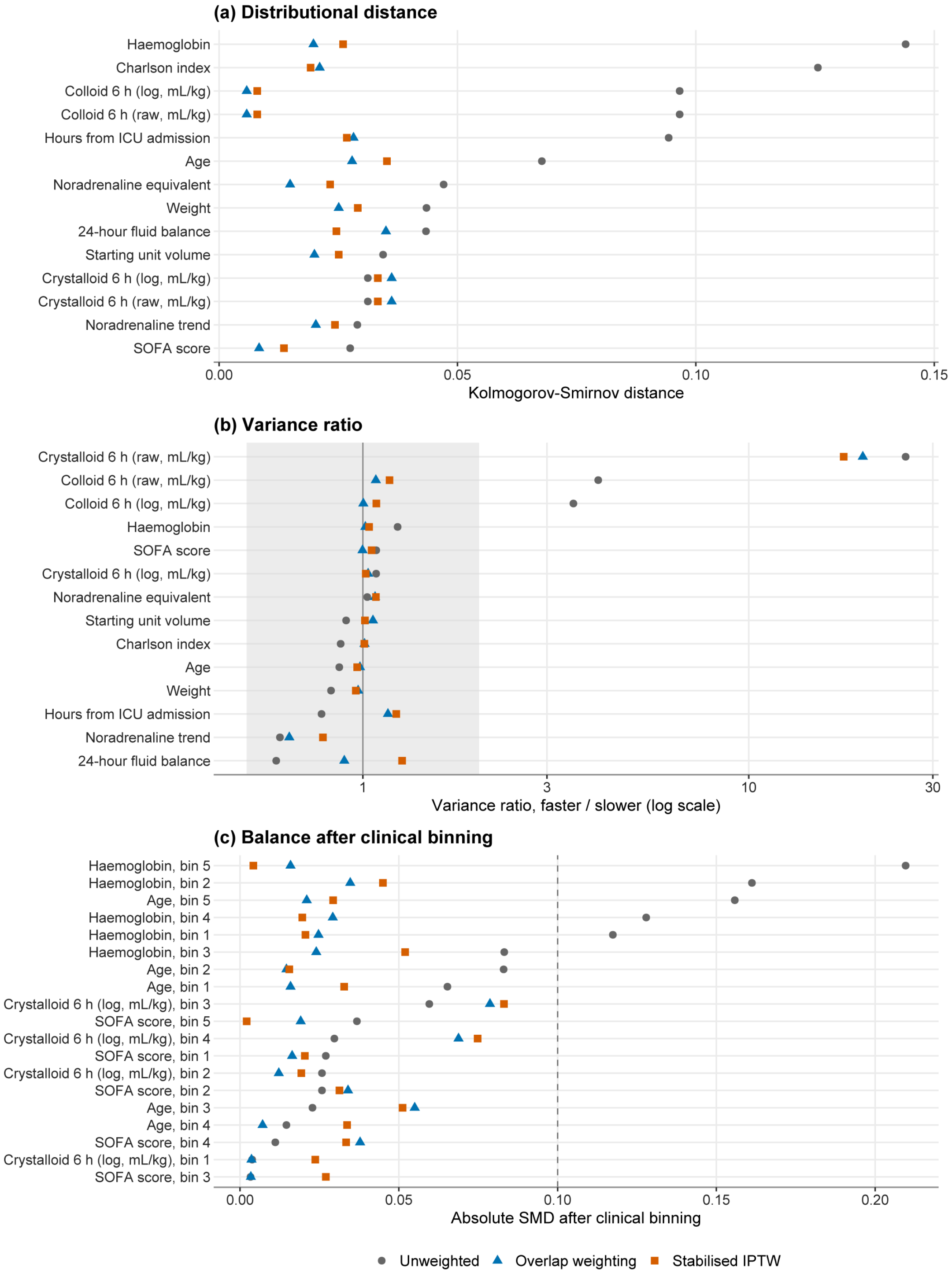

Figure S4. Distributional balance.

Kolmogorov–Smirnov distances, variance ratios against the Rubin reference range of 0.5–2.0, and standardised differences after clinical binning. Residual imbalance persists in the variance of crystalloid volume per kilogram, which enters the model log-transformed.

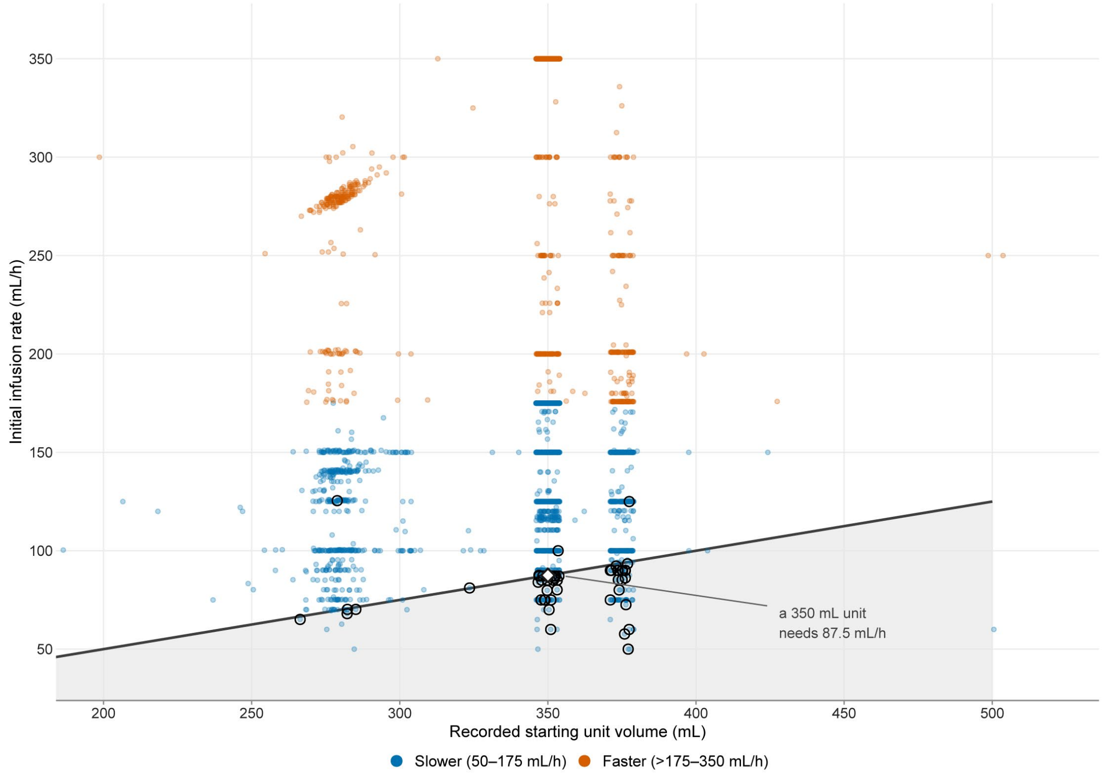

Figure S5. Whether the starting rate could complete the unit within four hours.

Each point is one transfusion episode, positioned by the volume recorded in the unit and the rate set on the first line. The diagonal is the rate at which a unit of that volume would finish in exactly four hours: 87.5 mL/h for a 350 mL unit, 62.5 mL/h for a 250 mL unit.

Supplementary Tables

Table S1. Respiratory-support level mapping.

Source	Recorded value	Level	Interpretation
S1_device	none	0	Room air (added as confirmatory evidence of non-escalation)
S1_device	aerosol-cool	1	Conventional oxygen
S1_device	face tent	1	Conventional oxygen
S1_device	high flow neb	1	Conventional oxygen
S1_device	medium conc mask	1	Conventional oxygen
S1_device	nasal cannula	1	Conventional oxygen
S1_device	non-rebreather	1	Conventional oxygen
S1_device	oxymizer	1	Conventional oxygen
S1_device	ultrasonic neb	1	Conventional oxygen
S1_device	vapomist	1	Conventional oxygen
S1_device	venti mask	1	Conventional oxygen
S1_device	high flow nasal cannula	2	HFNC
S1_device	bipap mask	3	NIV
S1_device	cpap mask	3	NIV
S1_device	endotracheal tube	4	IMV
S2_ce_223849	CPAP/PPS	3	Non-invasive ventilation mode (chartevents 223849)

S2_ce_229314	DuoPaP	3	Non-invasive ventilation mode (chartevents 229314)
S2_ce_229314	NIV	3	Non-invasive ventilation mode (chartevents 229314)
S2_ce_229314	NIV-ST	3	Non-invasive ventilation mode (chartevents 229314)
S2_vs_mode	Apnea Ventilation	4	Mode indicating invasive ventilation
S2_vs_mode	APV (cmv)	4	Mode indicating invasive ventilation
S2_vs_mode	CMV	4	Mode indicating invasive ventilation
S2_vs_mode	CMV/ASSIST	4	Mode indicating invasive ventilation
S2_vs_mode	CMV/ASSIST/AutoFlow	4	Mode indicating invasive ventilation
S2_vs_mode	CMV/AutoFlow	4	Mode indicating invasive ventilation
S2_vs_mode	CPAP	4	Mode indicating invasive ventilation
S2_vs_mode	CPAP/PSV	4	Mode indicating invasive ventilation
S2_vs_mode	CPAP/PSV+ApnPres	4	Mode indicating invasive ventilation
S2_vs_mode	CPAP/PSV+ApnVol	4	Mode indicating invasive ventilation
S2_vs_mode	MMV	4	Mode indicating invasive ventilation
S2_vs_mode	MMV/PSV	4	Mode indicating invasive ventilation
S2_vs_mode	MMV/PSV/AutoFlow	4	Mode indicating invasive ventilation
S2_vs_mode	PCV+	4	Mode indicating invasive ventilation
S2_vs_mode	PCV+/PSV	4	Mode indicating invasive ventilation

S2_vs_mode	PCV+Assist	4	Mode indicating invasive ventilation
S2_vs_mode	PRVC/AC	4	Mode indicating invasive ventilation
S2_vs_mode	PRVC/SIMV	4	Mode indicating invasive ventilation
S2_vs_mode	PSV/SBT	4	Mode indicating invasive ventilation
S2_vs_mode	SIMV	4	Mode indicating invasive ventilation
S2_vs_mode	SIMV/AutoFlow	4	Mode indicating invasive ventilation
S2_vs_mode	SIMV/PSV	4	Mode indicating invasive ventilation
S2_vs_mode	SIMV/PSV/AutoFlow	4	Mode indicating invasive ventilation
S2_vs_mode	VOL/AC	4	Mode indicating invasive ventilation
S2_vs_mode	CPAP/PPS	—	Non-invasive ventilation, emitted by source S2_vs_niv instead
S2_vs_mode	SPONT	—	Not classified (standby, spontaneous or ambient)
S2_vs_mode	Standby	—	Not classified (standby, spontaneous or ambient)
S2_vs_niv	mode=CPAP/PPS OR hamilton IN (NIV,NIV-ST,DuoPaP)	3	Non-invasive ventilation mode (ventilator settings); one row per record, matching the third arm of the locked implementation
S4_ventderiv	SupplementalOxygen	1	Conventional oxygen, interval record (added as

			confirmatory evidence)
S4_ventderiv	HFNC	2	High-flow nasal cannula, interval record
S4_ventderiv	NonInvasiveVent	3	Non-invasive ventilation, interval record
S4_ventderiv	InvasiveVent	4	Invasive mechanical ventilation, interval record

Table S2. Excluded and unascertained patients.

A: primary outcome classified (*n*=2671) versus unclassified (*n*=119).

Variable	Classified	Unclassified	SMD
Age (years)	67.6 (14.9)	62.5 (15.4)	-0.340
Female	47.2%	51.3%	+0.082
SOFA score at time zero	4.1 (2.8)	3.7 (2.7)	-0.158
Haemoglobin (g/dL)	7.6 (1.1)	7.4 (1.0)	-0.130
Charlson comorbidity index	5.9 (3.0)	5.8 (3.1)	-0.057
Heart failure	35.2%	26.9%	-0.179
Chronic kidney disease or ESRD	30.4%	23.5%	-0.154
Sepsis at time zero	49.9%	55.5%	+0.111
24-hour net fluid balance (mL)	3916.0 (8756.3)	4186.2 (10170.8)	+0.028
Noradrenaline equivalent (μg/kg/min)	0.0 (0.1)	0.0 (0.1)	-0.065
Hours from admission to time zero	44.6 (62.4)	59.5 (99.3)	+0.180
Faster strategy	35.9%	36.1%	+0.004

B: main cohort (*n*=2790) versus patients whose baseline respiratory level was indeterminate (*n*=879, excluded at the final step of cohort assembly)

Variable	Main cohort	Indeterminate baseline level	SMD
Age (years)	67.4 (14.9)	67.2 (15.5)	-0.010
Female	47.3%	49.9%	+0.052
SOFA score at time zero	4.1 (2.8)	4.3 (3.0)	+0.085
Haemoglobin (g/dL)	7.6 (1.1)	7.5 (1.1)	-0.070
Charlson comorbidity index	5.9 (3.0)	5.9 (2.9)	-0.001
Heart failure	34.8%	32.2%	-0.055
Chronic kidney disease or ESRD	30.1%	32.1%	+0.043
Sepsis at time zero	50.1%	52.4%	+0.046
24-hour net fluid balance (mL)	3927.5 (8819.4)	4573.0 (9645.7)	+0.070
Noradrenaline equivalent (μg/kg/min)	0.0 (0.1)	0.0 (0.1)	-0.016
Hours from admission to time zero	45.3 (64.4)	51.9 (81.1)	+0.090
Faster strategy	35.9%	32.7%	-0.070

Table S3. Outcome ascertainability.

A: the six outcome classification categories, by strategy

Category	Slower	Faster	Total	Percent
Definite non-event, observed to 12 h	1402	756	2158	77.35%
Assumed non-event, ward transfer before 12 h	221	149	370	13.26%
Definite event	81	50	131	4.70%
Unclassifiable respiratory records	71	36	107	3.84%
Death within 12 h	7	5	12	0.43%

Truncated observation	5	7	12	0.43%
Total	1787	1003	2790	100.00%

Note: death was ascertained hospital-wide, respiratory support only within intensive care. For patients discharged to a general ward before 12 hours, non-escalation is an assumption, not an observation; it can fail only by recording an escalation as a non-event. None of these patients re-entered intensive care within the window.

B: outcome classification under the two rules

Definition	Composition	*n*	Percent
Main	death + definite event + assumed non-event with ward transfer + complete observation	2671	95.73%
Ward-transfer-excluded	main rule with the ward transfers removed	2301	82.47%

C: completeness of the covariate observation window

Window	Slower	Faster	Difference(percentagepoints)
Complete 24-hour look-back	53.33%	61.71%	+8.39

Table S4. Alternative ascertainment thresholds.

Event counts across three ascertainment thresholds, three outcome definitions and three windows (whole cohort, N = 2,790).

Window	Ascertainment threshold	Any increase in level or death (KS-1)	Primary outcome	Invasive ventilation or death (KS-2)	N
12 hours after time zero	Strict	249	129	97	2790
12 hours after time zero	Main threshold	296	143	111	2790
12 hours after time zero	Permissive	312	160	111	2790
24 hours after time zero	Strict	368	208	150	2790
24 hours after time zero	Main threshold	426	224	167	2790

24 hours after time zero	Permissive	459	259	167	2790
6 hours after time zero	Strict	153	63	49	2790
6 hours after time zero	Main threshold	182	74	60	2790
6 hours after time zero	Permissive	199	90	60	2790

Note: the ascertainment threshold determines both whether an event is counted and whether the outcome can be classified under that threshold.

Table S5. Sensitivity analyses and E-values.

Batch	ID	Analysis	*n* (slower/faster)	Events (slower/faster)	Risk difference, percentage points (95% CI)	Risk ratio (95% CI)
—	Primary	Primary analysis, overlap weighting (reference)	1711/960	88/55	+1.65 (-0.41, +3.74)	1.33 (0.93, 1.92)
L2	S7	Stratified by record phenotype (exploratory) — template-ordered	455/587	13/35	+3.57 (+0.79, +6.49)	—
L2	S7	Stratified by record phenotype (exploratory) — other	1256/373	75/20	+0.91 (-2.19, +4.27)	—

		phenotypes				
L2	S7	Stratified by record phenotype (exploratory) — interaction	—	—	+2.66 (-1.89, +7.04)	—
L2	S8a	Outcome window changed to 6 hours	1711/960	44/30	+1.28 (-0.29, +2.96)	1.51 (0.90, 2.56)
L2	S8b	Outcome window changed to 24 hours	1711/960	142/80	+1.22 (-1.28, +3.64)	1.15 (0.86, 1.52)
L2	S9a	Missing outcomes all coded as non-events	1787/1003	88/55	+1.58 (-0.39, +3.56)	1.33 (0.92, 1.91)
L2	S9b	Missing outcomes all coded as events	1787/1003	164/98	+1.61 (-0.99, +4.33)	1.18 (0.90, 1.53)
L2	S12a	NEGATIVE CONTROL (pre-specified criterion, any increase in level): 12 h before	948/600	77/44	-1.57 (-4.83, +1.71)	0.83 (0.55, 1.23)

		time zero				
L2	S12b	NEGATIVE CONTROL (pre-specified criterion, any increase in level): 6 h before time zero	1160/690	58/32	-0.93 (-3.19, +1.21)	0.82 (0.49, 1.29)
L2	S12c	Secondary negative control: new positive culture, 72 h to 7 days	1787/1003	171/87	+0.85 (-1.71, +3.29)	1.10 (0.82, 1.43)
L2	S16	Escalation only, with death treated as a competing event	1711/960	82/52	+1.59 (-0.45, +3.70)	1.34 (0.92, 1.94)
L2	S24	Ward transfers excluded rather than treated as non-events	1490/811	88/55	+2.07 (-0.31, +4.55)	1.36 (0.95, 1.95)
L2	S25a	Strict ascertainm	1701/953	79/50	+1.41 (-	1.31 (0.89,

		ent threshold (analysis population varies)			0.56, +3.41)	1.91)
L2	S25b	Permissive ascertainment threshold (analysis population varies)	1711/960	99/61	+1.58 (-0.59, +3.82)	1.28 (0.90, 1.82)
L1L4	S1	Exposure redefined using first-record originalrate (regrouped; propensity score refitted)	1785/886	93/50	+1.71 (-0.47, +3.89)	1.35 (0.92, 1.94)
L1L4	S2	Buffer cut-points, ≤150 vs >200 mL/h (560 patients in the buffer excluded)	1367/775	75/45	+1.88 (-0.72, +4.52)	1.36 (0.89, 2.11)
L1L4	S18	Comorbidity index removed from the propensity	1711/960	88/55	+1.64 (-0.40, +3.77)	1.33 (0.93, 1.90)

		score model				
L1L4	S19	Alternative sepsis definition	1711/960	88/55	+1.65 (-0.40, +3.75)	1.33 (0.93, 1.91)
L1L4	S21	Fluid balance rescaled by observation time (mL/kg/h)	1711/960	88/55	+1.65 (-0.38, +3.76)	1.33 (0.94, 1.91)
L1L4	S26	Look-back-window completeness added to the propensity score model	1711/960	88/55	+1.65 (-0.41, +3.75)	1.33 (0.93, 1.92)
L1L4	S6a	Stabilised inverse-probability weighting, untruncated	1711/960	88/55	+2.08 (-0.22, +4.68)	1.42 (0.96, 2.04)
L1L4	S6b	Stabilised inverse-probability weighting, truncated at 1st/99th	1711/960	88/55	+2.05 (-0.22, +4.33)	1.41 (0.96, 1.99)

		percentiles				
L1L4	S6c	Stabilised inverse-probability weighting, truncated at 2.5th/97.5th percentiles	1711/960	88/55	+1.84 (-0.26, +4.13)	1.37 (0.95, 1.94)
L1r	S13	Restricted to the six units with substantial representation of both strategies	1671/940	88/54	+1.57 (-0.50, +3.70)	1.31 (0.91, 1.88)
L1r	S14	Starting unit volume within the technical range 250-450 mL	1700/957	88/55	+1.65 (-0.42, +3.75)	1.33 (0.92, 1.91)
L1r	S15	Restricted below the 90th percentile of prior crystalloid (11.465 mL/kg)	1546/860	76/48	+2.16 (-0.04, +4.42)	1.48 (0.99, 2.18)
L1r	S17	Starting unit	1263/732	61/40	+1.36 (-	1.27 (0.83,

		volume at the two modal values (350 ± 1 and 375 ± 1 mL)			1.03, +3.87)	1.93)
L1r	S20	Restricted to patients with a complete 24-hour covariate look-back window	911/593	43/22	+0.05 (-2.48, +2.47)	1.01 (0.56, 1.72)
L1r	S22	Slower group restricted to units whose whole-episode hang time was within 4 hours	1661/960	85/55	+1.69 (-0.36, +3.79)	1.34 (0.94, 1.93)
L0	S4_P3	Cohort P3: 4 h plus the broader bleeding exclusion	1694/935	86/49	+1.38 (-0.68, +3.46)	1.29 (0.88, 1.88)
L0	S5	Broadest bleeding exclusion, adding admission	1462/849	71/42	+1.42 (-0.68, +3.54)	1.32 (0.87, 1.97)

		bleeding diagnoses				
L0_P4	S4_P4	Cohort P4: 2 h plus the broader bleeding exclusion	1963/1092	107/64	+1.37 (-0.67, +3.36)	1.26 (0.89, 1.73)
L3	S23	Inverse-probability-of-observation weighting (overlap weight × observation weight)	1711/960	88/55	+1.60 (-0.42, +3.71)	1.32 (0.92, 1.91)

— Post-hoc diagnostics (not among the pre-specified analyses) —

L2	S12a-m	NEGATIVE CONTROL matched to the primary outcome definition, 12 h (post hoc)	948/600	23/12	-0.80 (-2.38, +0.66)	0.69 (0.28, 1.37)
L2	S12b-m	NEGATIVE CONTROL matched to the primary outcome definition, 6 h (post hoc)	1160/690	11/5	-0.51 (-1.50, +0.39)	0.54 (0.09, 1.64)
L2	S25a_fixN	Strict	1711/960	79/50	+1.39 (-0.57,	1.31 (0.89,

		ascertainment threshold, population fixed at N = 2,671 (post-hoc diagnostic)			+3.39)	1.90)
L2	S25b_fixN	Permissive ascertainment threshold, population fixed at N = 2,671 (post-hoc diagnostic)	1711/960	99/61	+1.58 (-0.59, +3.82)	1.28 (0.90, 1.82)

— E-value (S10) —

Outcome	Risk ratio (95% CI)	E-value, point estimate	E-value, confidence limit	Note
Primary: escalation of advanced respiratory support or death (12 h)	1.33 (0.93, 1.92)	2.00	1.00	Interval includes the null: no unmeasured confounding at all is required
Key secondary 1: any increase in respiratory support or death (12 h)	1.22 (0.96, 1.55)	1.74	1.00	Interval includes the null: no unmeasured confounding at all is required
Key secondary 2: invasive mechanical ventilation or	1.71 (1.16, 2.58)	2.81	1.60	Bound nearer the null = 1.165

death (12 h)				

Table S6. Full cohort characteristics.

The first three value columns are unweighted; the next three are after overlap weighting (OW). The final column describes the 226 episodes above 350 mL/h, which are outside the strategy comparison. SMD, standardised mean difference.

Variable	Slower	Faster	SMD	Slower (OW)	Faster (OW)	SMD (OW)	>350 mL/h
— Exposure, fixed at time zero —							
First recorded infusion rate (mL/h)	127.5 (31.6)	286.0 (66.7)	—	132.6 (32.1)	277.1 (68.4)	—	476.8 (211.3)
— Baseline covariate: the unit —							
Recorded starting unit volume (mL)	338.0 (37.5)	339.5 (35.6)	0.041	339.7 (35.8)	339.7 (36.9)	0.000	361.2 (30.4)
— Demographics and admission —							
Age (years)	68.1 (15.3)	66.1 (14.2)	-0.139	66.4 (14.8)	66.4 (14.7)	-0.000	67.0 (13.9)
Female	48.8%	44.8%	-0.081	46.1%	46.1%	-0.000	46.5%
Weight (kg)	79.2 (22.0)	79.6 (20.0)	0.021	79.6 (20.9)	79.6 (20.6)	0.000	83.2 (24.5)
Hours from intensive care admission to time zero	46.0 (67.1)	43.9 (59.3)	-0.034	44.5 (62.0)	44.5 (66.8)	-0.000	44.5 (73.2)
First intensive care stay	76.2%	78.7%	0.060	77.2%	77.2%	0.000	75.2%
Started at	29.2%	28.6%	-0.013	29.6%	29.6%	0.000	31.9%

night							
Started at a weekend	29.9%	26.9%	-0.067	28.4%	28.4%	0.000	31.4%
— Severity of illness —							
SOFA score at time zero	4.12 (2.72)	4.07 (2.83)	-0.018	4.10 (2.86)	4.10 (2.86)	0.000	3.96 (2.53)
Sepsis at time zero	51.9%	47.1%	-0.096	48.0%	48.0%	0.000	48.7%
Haemoglobin (g/dL)	7.46 (1.08)	7.73 (1.20)	0.241	7.62 (1.17)	7.62 (1.18)	-0.000	7.67 (1.13)
Noradrenaline equivalent (μg/kg/min)	0.02 (0.08)	0.02 (0.08)	0.025	0.02 (0.08)	0.02 (0.08)	-0.000	0.03 (0.09)
Charlson comorbidity index	6.19 (3.02)	5.46 (2.83)	-0.249	5.71 (2.90)	5.71 (2.92)	-0.000	5.49 (3.00)
Heart failure	40.0%	25.6%	-0.309	29.9%	29.9%	0.000	27.4%
Chronic kidney disease or end-stage renal disease	32.1%	26.5%	-0.122	27.8%	27.8%	0.000	27.9%
Receiving renal replacement therapy	5.9%	6.6%	0.029	6.8%	6.8%	0.000	7.1%
— Volume status —							
24-hour net fluid balance (mL)	4149.8 (9532.2)	3531.5 (7358.7)	-0.073	3914.5 (8904.0)	3914.5 (8423.7)	-0.000	4603.9 (9824.5)
Crystalloid in the 6 h	4.17 (8.59)	5.91 (43.37)	0.055	4.61 (9.77)	5.94 (43.44)	0.042	4.53 (7.02)

before time zero (mL/kg)							
Colloid in the 6 h before time zero (mL/kg)	0.17 (1.13)	0.66 (2.29)	0.270	0.34 (1.56)	0.34 (1.62)	0.002	0.52 (2.04)
Loop diuretic before time zero	31.6%	35.5%	0.082	31.9%	31.9%	0.000	24.8%
— Baseline respiratory support level —							
0 Room air or no oxygen	33.8%	29.3%	-0.097	31.6%	31.6%	-0.000	30.1%
1 Conventional oxygen	61.7%	66.1%	0.091	63.9%	63.9%	0.000	67.3%
2 High-flow nasal cannula	2.8%	3.0%	0.012	2.8%	2.8%	0.000	0.9%
3 Non-invasive ventilation	1.7%	1.6%	-0.007	1.7%	1.7%	0.000	1.8%
— Intensive care unit type —							
Cardiac Vascular Intensive Care Unit (CVICU)	13.0%	39.6%	0.634	28.0%	28.0%	0.000	20.4%
Coronary Care Unit (CCU)	19.0%	4.7%	-0.455	7.8%	7.8%	0.000	8.0%
Intensive Care Unit	0.1%	0.1%	-0.004	0.1%	0.1%	0.000	0.0%

(ICU)							
Medical Intensive Care Unit (MICU)	22.9%	15.6%	-0.187	21.0%	21.0%	0.000	19.5%
Medical/Surgical Intensive Care Unit (MICU/SICU)	30.4%	11.0%	-0.494	17.6%	17.6%	-0.000	7.1%
Neuro Intermediate	1.3%	1.1%	-0.018	1.4%	1.4%	0.000	0.9%
Neuro Stepdown	0.3%	0.3%	0.004	0.4%	0.4%	0.000	0.4%
Neuro Surgical Intensive Care Unit (Neuro SICU)	0.8%	0.3%	-0.066	0.5%	0.5%	0.000	1.3%
PACU	0.1%	0.1%	0.016	0.1%	0.1%	0.000	0.0%
Surgery/Trauma	0.1%	0.0%	-0.033	0.0%	0.0%	-0.000	0.0%
Surgery/Vascular/Intermediate	0.0%	0.1%	0.045	0.0%	0.0%	0.000	0.0%
Surgical Intensive Care Unit (SICU)	8.3%	18.2%	0.295	15.6%	15.6%	0.000	24.8%
Trauma SICU (TSICU)	3.8%	9.0%	0.213	7.5%	7.5%	0.000	17.7%

— Admission type —							
Elective	12.4%	24.4%	0.315	18.7%	18.7%	0.000	21.7%
Emergency	74.0%	62.4%	-0.250	67.6%	67.6%	-0.000	65.9%
Observation	13.7%	13.2%	-0.014	13.7%	13.7%	0.000	12.4%
— Post-time-zero infusion process (not baseline covariates; not balanced) —							
Whole-episode hang time (min)	157.8 (42.9)	75.9 (23.6)	—	151.7 (42.2)	78.7 (24.9)	—	51.3 (14.8)
— Descriptive variables outside the propensity score model —							
Lactate measured	32.6%	31.8%	-0.016	31.8%	32.1%	0.006	33.2%
Complete 24-hour look-back window	53.3%	61.7%	0.170	56.7%	57.1%	0.006	50.4%

Table S7. Transitions for the first key secondary outcome.

Transition	Slower	Faster	Total
0→1	91	61	152
1→4	36	31	67
0→4	11	11	22
1→3	17	4	21
Death without an increase in level	6	3	9
3→4	4	4	8
1→2	6	1	7
2→4	4	1	5
0→3	4	0	4
2→3	1	0	1

Table S8. Other secondary outcomes.

Outcome	Type	*n* (slower/faster)	Events (slower/faster)	Weighted, slower	Weighted, faster	Difference (95% CI)
Newly started intravenous loop diuretic, 0–12 h (restricted to patients on no diuretic in the 24 h before time zero)	risk difference	1371/712	168/80	12.10%	9.52%	-2.58 pp (-5.58, +0.50)
Acute kidney injury within 48 h (KDIGO, creatinine or urine output)	risk difference	1777/994	1102/641	61.60%	63.28%	+1.68 pp (-2.12, +5.71)
28-day mortality (exploratory)	risk difference	1787/1003	334/111	15.17%	13.72%	-1.45 pp (-4.10, +1.30)
Cumulative fluid balance, 0–24 h after time zero (mL)	mean difference	1787/1003	—	2628	2638	+10 mL (-654, +666)
Length of intensive	weighted median	1787/1003	—	median 46.5 (IQR 23.1-	median 45.5 (IQR 22.3-	descriptive; no interval

care stay from time zero (hours)	(IQR)			105.6)	97.7)	computed
Alive discharge from intensive care, cumulative incidence at 24 h	cumulative incidence difference	1787/1003	—	25.93%	25.95%	+0.02 pp (-3.41, +3.69)
Alive discharge from intensive care, cumulative incidence at 48 h	cumulative incidence difference	1787/1003	—	49.38%	50.17%	+0.79 pp (-3.24, +4.89)
Alive discharge from intensive care, cumulative incidence at 72 h	cumulative incidence difference	1787/1003	—	62.32%	64.38%	+2.06 pp (-1.98, +5.90)
Alive discharge from intensive care, cumulative incidence at 168 h	cumulative incidence difference	1787/1003	—	79.43%	81.27%	+1.84 pp (-1.34, +5.13)

Death in intensive care, cumulative incidence at 24 h	cumulative incidence difference	1787/1003	—	0.63%	0.77%	+0.13 pp (-0.57, +0.88)
Death in intensive care, cumulative incidence at 48 h	cumulative incidence difference	1787/1003	—	1.58%	1.61%	+0.03 pp (-1.06, +1.08)
Death in intensive care, cumulative incidence at 72 h	cumulative incidence difference	1787/1003	—	2.26%	2.25%	-0.01 pp (-1.24, +1.27)
Death in intensive care, cumulative incidence at 168 h	cumulative incidence difference	1787/1003	—	4.17%	4.00%	-0.17 pp (-1.80, +1.56)

Table S9. Outcome validity by level of support reached.

A. 28-day in-hospital mortality by 12-hour outcome status (n = 2,671)

Outcome	Events/n with outcome	% with outcome	Events/n without	% without	Difference (pp)
Primary outcome (escalation of advanced respiratory support or death, 12 h)	51/143	35.7	248/2528	9.8	+25.9

Invasive mechanical ventilation or death, 12 h	46/111	41.4	253/2560	9.9	+31.6
Any increase in respiratory-support level or death, 12 h	63/296	21.3	236/2375	9.9	+11.3
Respiratory escalation only (12-hour deaths retained)	42/134	31.3	257/2537	10.1	+21.2

B. 28-day in-hospital mortality by the level of respiratory support reached

(patients who died within the 12-hour window are excluded, so the comparison is not circular)

Level reached by 12 h	Deaths/*n*	28-day mortality (%)
Reached invasive mechanical ventilation (level 4)	34/99	34.3
Escalation stopped at high-flow or non-invasive support (level 2-3)	5/32	15.6
No escalation	236/2375	9.9

Table S10A. Included and non-included ICU RBC recipients.

Characteristic	Included (n = 2790)	Not included (n = 12,255)
Age at index ICU admission, years	69 [58–78]	68 [56–78]
First RBC record after ICU admission, hours	28.98 [11.22, 50.24]	9.75 [2.72, 30.56]
Sex		
Female	1321 (47.3%)	5413 (44.2%)
First care unit		
Cardiac Vascular Intensive Care Unit (CVICU)	629 (22.5%)	3082 (25.1%)
Coronary Care Unit (CCU)	387 (13.9%)	722 (5.9%)
Intensive Care Unit (ICU)	3 (0.1%)	7 (0.1%)
Medical Intensive Care Unit (MICU)	565 (20.3%)	3006 (24.5%)
Medical/Surgical Intensive Care Unit (MICU/SICU)	653 (23.4%)	1850 (15.1%)
Medicine	0 (0.0%)	3 (0.0%)
Neuro Intermediate	34 (1.2%)	94 (0.8%)
Neuro Stepdown	8 (0.3%)	37 (0.3%)
Neuro Surgical Intensive Care Unit (Neuro SICU)	17 (0.6%)	111 (0.9%)
PACU	2 (0.1%)	11 (0.1%)
	1 (0.0%)	0 (0.0%)

Surgery/Trauma		
Surgery/Vascular/Intermediate	1 (0.0%)	41 (0.3%)
Surgical Intensive Care Unit (SICU)	332 (11.9%)	1495 (12.2%)
Trauma SICU (TSICU)	158 (5.7%)	1796 (14.7%)
Hospital admission type		
DIRECT EMER.	130 (4.7%)	563 (4.6%)
DIRECT OBSERVATION	2 (0.1%)	4 (0.0%)
ELECTIVE	130 (4.7%)	565 (4.6%)
EU OBSERVATION	1 (0.0%)	7 (0.1%)
EW EMER.	1165 (41.8%)	5808 (47.4%)
OBSERVATION ADMIT	373 (13.4%)	1338 (10.9%)
SURGICAL SAME DAY ADMISSION	336 (12.0%)	1295 (10.6%)
URGENT	653 (23.4%)	2675 (21.8%)
Patient anchor-year group		
2008 - 2010	954 (34.2%)	4271 (34.9%)
2011 - 2013	586 (21.0%)	2864 (23.4%)
2014 - 2016	585 (21.0%)	2487 (20.3%)
2017 - 2019	517 (18.5%)	1982 (16.2%)
2020 - 2022	148 (5.3%)	651 (5.3%)

Values are median [interquartile range] or number (%). The included group comprises the study cohort. Demographic and admission descriptors refer to index ICU admission, not an imputed transfusion-time clinical baseline. No listed variable was missing in either group. No outcome comparison, hypothesis testing or reweighting was performed. This comparison is post hoc. Admission-type and care-unit labels retain the source database categories. Patient anchor-year group is the source field anchor_year_group: the real-year range associated with the patient's anchor year, not an exact index-admission calendar period.

Table 2 admission-type categories: Emergency = DIRECT EMER., EW EMER. and URGENT;

Elective = ELECTIVE and SURGICAL SAME DAY ADMISSION (130 + 336 = 466 included patients); Observation = AMBULATORY OBSERVATION, DIRECT OBSERVATION, EU OBSERVATION and OBSERVATION ADMIT.

Table S10B Sequential exclusions from 15,045 ICU RBC recipients.

Criterion applied at this step	Excluded at step	Remaining
No eligible positive-rate mL/hour record	2	15,043
Initial recorded rate <50 mL/h	40	15,003
RBC initiation <4 h after ICU admission	3,902	11,101
High-specificity bleeding/context indicators (union)	5,075	6,026
Initial recorded rate >350 mL/h	595	5,431
Baseline invasive mechanical ventilation	1,762	3,669
Indeterminate baseline respiratory support	879	2,790

Step counts are mutually exclusive because exclusions are sequential. They sum to 12,255. The six bleeding/context indicators can overlap within their union. Patients outside the target-trial eligibility criteria are not described as eligible patients lost to follow-up.
